# Smartphone and tablet use pattern in children up to 5 years old in Spain: a cross-sectional study *

**DOI:** 10.1590/1518-8345.7276.4377

**Published:** 2024-11-22

**Authors:** Sonia de Paz-Cantos, Adrián González-Marrón, Cristina Lidón-Moyano, Maria Cerrato-Lara, Ana Díez-Izquierdo, Jose M Martínez-Sánchez

**Affiliations:** ^1^ Universitat Internacional de Catalunya, Barcelona, Sant Cugat del Vallès, Spain.; ^2^ Universidad Internacional de La Rioja, Logroño, Spain.

**Keywords:** Electronic Devices, Mobile Applications, Preschool Child, Health Promotion, Smartphone, Tablet

## Abstract

to characterize the smartphones and tablets use pattern in a sample of children up to 5 years old and assess with which variables it is associated.

cross-sectional study of 410 parents of children up to 5 years of age. Data were obtained from an online questionnaire. Questions about the time and pattern of daily use of smartphones and tablets were collected. We calculated the overall percentage (and 95% confidence interval) and median (and interquartile range) of each device’s use time and according to the variables associated with the child, and the respondent’s characteristics.

an estimated 44.7% (95% CI: 37; 51) of children used both smartphones and/or tablets daily. The median use time for smartphones and tablets separately was 30 minutes per day for smartphones (8.6-38.6) and 30 (17.1- 60.0) for tablet. In children who have free access to the devices, 11.6% (95% CI: 6; 16) use these devices up to 30 minutes after waking up on weekdays, 15.4% (95% CI: 10; 21) during weekends and 14.0% (95% CI: 8; 19) use smartphone and/or tablet up to 1 hour before going to bed.

more than 4 out of 10 children in our sample used smartphones and/or tablets daily. Community nursing may be a viable avenue for implementing educational programs focused on promoting healthy practices in children’s electronic device usage given the pattern of excessive screen use in the pediatric population.

## Highlights

**(1)** Separate screen usage according to location and usage.

**(2)** Parent’s educational level influences children’s mobile device usage time.

**(3)** Pediatric recommendations should be followed.

**(4)** Politicians, nurses and teachers must work together.

**(5)** More than 4 out of 10 children in our sample use smartphones and tablets daily.

## Introduction

 Screen devices are ubiquitous and used for multiple tasks, including entertainment, communication, and learning. Children and adolescents are constantly exposed to a wide range of screen devices, including smartphones, tablets and smartwatches, among others) ^(^ 1 ^-^ 2 ^)^ . Consequently, smartphones and tablets use time has increased in the last years ^(^ 3 ^)^ . Besides, the indiscriminate use of screens has also invaded homes, causing children to start using digital media at increasingly younger ages, between 12 and 24 months of age ^(^ 4 ^)^ . For instance, in South Korea, 12.2% of children start using digital media prior to the age of 6 months to 4 years; most 3- and 4-year-olds regularly access their parents’ phones without any assistance according to a sample of children from Philadelphia ^(^ 5 ^)^ . 

 The smartphone use has positive and negative health effects ^(^ 6 ^)^ . On the one hand, excessive use of smartphone and tablet (use the screen more than 1 hour per day) is associated with deleterious health outcomes in preschool children. There is evidence that screen time is associated with sleep (mainly with reduced duration) and with physical, mental, and psychosocial functioning of infants and toddlers ^(^ 7 ^-^ 8 ^)^ . In addition, obesity is one of the best-documented outcomes associated with screen exposure ^(^ 9 ^-^ 10 ^)^ . On the other hand, the use of screens can have positive effects such as quick access to information, increased communication, even educational learning ^(^ 6 ^)^ . Parents might introduce screen devices to help children with schoolwork. Also, there is preliminary evidence that interactive “learning to read” applications and e-books can promote early literacy through letter, picture, and word recognition practice ^(^ 11 ^)^ . 

 Given the growing trend in the use of smartphones and tablets at increasingly younger ages, it is important to make efforts to prevent the negative effects of screen use from a nursing perspective. The school nursing’s role could be very important in promoting healthy use of screens ^(^ 12 ^)^ . Nurses are the health professionals who have the greatest contact with families in pediatric primary care. Therefore, they could lead programs to promote the good use of technology and even design interventions to reduce the misuse of smartphones. 

 Different institutions have made recommendations for their use. According to the Ministry of Health, Social Services and Equality ( *Ministerio de Sanidad, Servicios Sociales e Igualdad* : MSSSI, from Spain), children under 3 years of age should not be exposed to screens, and screen time of more than 1 hour per day is considered excessive among children aged 2-4 years ^(^ 13 ^)^ . Similarly, the American Academy of Pediatrics (AAP) recommends that children aged 18 to 24 months should only be exposed to high-quality applications (for example: Duolingo) and that children aged 3 to 5 years should be limited to 1 hour of screen time with high-quality programs in the presence of parents ^(^ 14 ^)^ , recommendation recently adopted by the Government of Catalonia (Spain) ^(^ 15 ^)^ . Thus far, there are no specific recommendations of smartphone and tablet use time. Besides, the Spanish National Health Survey reports exposure to screen time in general, without differentiation by device ^(^ 13 ^,^ 16 ^)^ . Therefore, the aim of this study was to characterize smartphones and tablets use pattern in a sample of children up to 5 years old and assess with which variables it is associated. 

## Method

### Study design

A cross-sectional study was conducted from March 2021 to March 2022. This research is reported following the STROBE (Strengthening the Reporting of Observational Studies in Epidemiology) guideline.

### Study setting

 We used baseline data of the Smart Screen Health project, which aims to promote healthy use of screen devices in early childhood. From the Kenko Lab (a study center for healthy screen use during childhood in Spain), we are carrying out several studies to generate scientific evidence and solutions that help families generate good habits with technology and find ways to improve child development ^(^ 17 ^)^ . 

 Data were collected at the *Hospital General de Catalunya* , a second level reference medical center located in the autonomous community of Catalunya (Spain). The researchers distributed in the main pediatric ward of the *Hospital General de Catalunya* (HUGC) (Sant Cugat del Vallès, Barelona, Spain) leaflets which included the quick response (QR) code linked to a questionnaire to parents who were attending the pediatric consultations. In addition, two posters were hung in the waiting room of the HUGC with the QR to directly access the questionnaire. Besides, the accounts of a pediatrician influencer “dospediatrasencasa” (Instagram and Twitter) were also used to disseminate the questionnaire to parents outside the HUGC. 

### Population and sample

We recruited a parents’ sample of Spanish children under 5 years of age. A non-probabilistic, consecutive sample was formed by parents attended at the study sitting and who met the following eligibility criteria: were parents who had children aged 3 months up to 5 years old. A total of 461 responses were obtained. Out of these 461 responses, 51 responses that had errors (non-plausible values) in the variables of children’s age and parents’ age were eliminated. Specifically, 6 children and 45 parents were eliminated. After data cleaning, the final sample included 410 responses.

### Sampling size

The sample size was calculated with the simple random sampling formula (N = ([Z-p-(1-p)]/e)2) for an estimated prevalence (p) of 50% (prevalence maximizing the sample size), a 95% confidence level (= 0.05; Z = 1.96) and a precision or error of 5% (e = 0.05). According to that, 384 subjects were required.

### Data collection instrument

An online non-validated survey was used to collect responses from parents with children between 3 months and 5 years of age in Spain. The online questionnaire in Spanish had 22 questions and was implemented in Google Forms. The questionnaire included health, sociodemographic, and smartphone, tablet, and TV exposure related questions.

### Study variables

The study variables included the outcomes below:

#### Smartphone and/or tablet use

The smartphone and/or tablet estimation use was obtained separately through two questions: “Approximately, how much time does your child spend in front of the following devices daily (Monday through Friday)?” and “Approximately, how much time does your child spend in front of the following devices on weekends (Saturday and Sunday)?”. Both were requested in 30-minute slots (“0 minutes”, “30 minutes”, “1 hour”, “1 hour and 30 minutes”, “2 hours”, “2 hours and 30 minutes”, “3 hours”, “3 hours and 30 minutes”, “4 hours or more”). “4 hours or more” was transformed to 4 hours (240 minutes) and it was assumed that children exposed to 4 hours or more of screen time watched exactly 4 hours. Children of parents who reported at least 30 minutes of smartphone and tablet use were considered users. Otherwise, children who reported 0 minutes of use were considered non-users.

#### Smartphone and/or tablet daily use time

Daily smartphone and/or tablet use was assessed through the questions mentioned above in the section of smartphone and/or tablet use. To obtain the mean daily time use, the weighted mean of the responses to both questions from Monday to Friday and Saturday to Sunday was performed. To estimate each device’s daily time use, the responses to each of the two questions were weighted by multiplying them by 5 and by 2, respectively. These results were then summed and divided by 7.

#### Free access to any smartphone and/or tablet of any family member

To estimate free access to smartphone and/or tablet, three questions were asked: “Does your child have free access to any smartphone or tablet of any family member?”(“yes”, “no”); “To which of these devices?”(“smartphone”, “tablet”); “Does your child consider the smartphone/tablet to be his/her own?”.

#### Type of application use and exclusive use application

To describe the used applications’ type and the exclusive use application type, three questions were asked: “What type of application does your child frequently use? (”videos”, “games”, “educational”, “social networking”, “music”, “other”, “not use”, “don’t know”/“no answer”); “Do you have any applications on your smartphone for your child’s exclusive use?” (“yes”, “no”, “don’t know/ don’t answer”); for those answering “yes” in the previous, parents could respond “Which one?” (“videos”, “games”, “educational”, “social networking”, “music”, “other”, “not use”, “don’t know”/“no answer”). In addition, these questions were also used for those parents who responded that their children were exposed to the display device: Does your child make use of the smartphone or tablet upon waking up? (“yes”, “no”). Does your child make use of the smartphone or tablet before going to bed? (“yes”, “no”). Two questions were used to estimate the time spent using the smartphone or tablet after waking up on weekdays and weekends: “How long does it take your child to ask for/use the smartphone after waking up on school days (Monday-Friday)?”, “How long does it take your child to ask to use the smartphone after waking up on weekends (Saturday-Sunday) or holidays?”, both with the possible answers (“no request”, “5 minutes or less”, “6 to 15 minutes”, “16 to 30 minutes”, “31 minutes to an hour”, “more than 1 hour”, “don’t know”/“no answer”). For the study of smartphone or tablet use before going to bed: “Does your child use smartphone or tablet before going to bed?” (“yes”, “no”, “don’t know”/“no answer”), “How long before?” (“30 minutes before”, “1 hour before”, “more than 1 hour before”, “don’t know/”no answer”).

### Covariates

#### Variables associated with the child

Gender (“male”, “female”); age (“≤ 2years old”, “3-5 years old”), having siblings (“yes”, “no”); and having older siblings (“yes”, “no”). For those having siblings, it was also collected if the siblings were older.

#### Variables associated with the respondent

Relationship of the respondent with the child (“mother”, “father”); age (“≤ 35 years”, “>35 years”) and educational level (“non-University”, “University”).

### Statistical analysis

The percentage of children using smartphone and/or tablet was calculated. The median and interquartile range (IQR) of the daily time use per device (smartphone and/or tablet) was calculated for each user of each device. Median and IQR of usage time were calculated due to violation of the normality assumption. Also, we calculated the overall percentage of children using smartphone and/or tablet (n=183) with free access to device, and according to covariates. The overall percentages of application’s type application and of parents’ possession of exclusive applications for their children’s use, stratified by age, were calculated. Additionally, the percentage of exposure to smartphone and/or tablet upon waking up and before bedtime was calculated overall and stratified by age. To study the association between outcomes and covariates, Mann-Whitney, Chi-Square, and Z-score tests were carried out. The statistical program used to perform the statistical analyses was R-4.1.1. The level of significance was set at 0.05.

### Ethical aspects

Before starting the study, participants had access to the information sheet and the informed consent document, which they had to fill out. To agree to complete the survey, participants filled out an online informed consent, as all data are treated anonymously and only for scientific purposes. The present study was approved by the Research Ethics Committee of the HUGC, Barcelona (2021/09-PED-HUGC).

## Results

**Figure 1 -:**
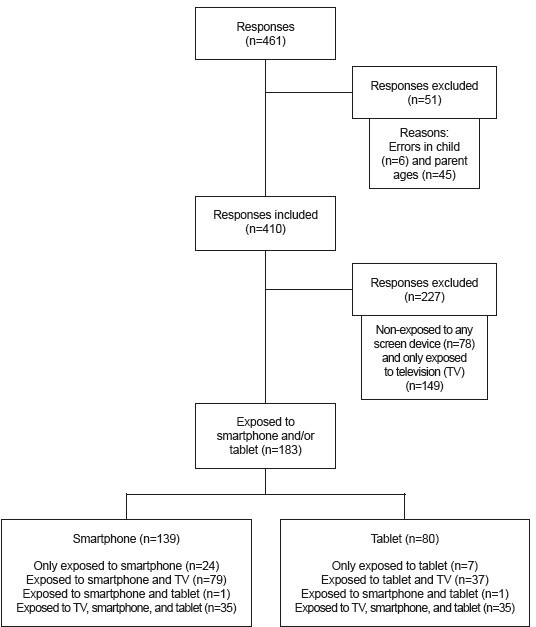
Flow chart of children exposed and non-exposed to screen devices in the sample

 The final sample included information of 410 children, of whom 44.7% were exposed to smartphone and/or tablet ( Figure 1 ). 57.0% of the children were females, 66.8% were under 3 years of age, 86.8% of the parents had university studies, 38.5% of the children had siblings, and of those, 65.8% had older siblings. 

 Of the 410 children, 33.9% (95% CI: 29; 38) used smartphone, 19.6% (95% CI: 15; 23) used tablet and 44.7% (95% CI: 37; 52) used both devices. The median (Q1-Q3) daily time use of smartphone was 30.0 (8.6-38.6) minutes and 30.0 (17.1-60.0) minutes for tablet. The probability of use of smartphones was significantly associated with the parents’ educational level (p-value <0.001) and the child’s age (p-value 0.018). In the case of the tablet, the probability of use was associated with the age of the child, having siblings, and the respondents’ age (p-value <0.001) ( Table 1 ). 

**Table 1 -:** Percentage (%) and median (and interquartile range) time of use (in minutes) of smartphone and/or tablet according to sociodemographic variables of children and family. Spain, 2021-2022

	**Smartphone(n=139)**			**Tablet(n=80)**		**Smartphone(n=139)**			**Tablet(n=80)**	
	**Percentage of use (%)** *					**Time of use (minutes per day)** ^‡^				
	**n**	**%( 95% CI)**	**p-value**	**%(95% CI)**	**p-value**	**n** ^†^	**median (Q1-Q3)**	**p-value**	**median (Q1-Q3)**	**p-value**
**Overall**	410	33.9(29; 38)		19.6(15; 23)		183	30.0(8.6-38.6)		30.0(17.1-60.0)	
**Child’s gender**			0.551		0.770			0.056		0.856
Male	234	35.8(30; 41)		18.8(14; 24)		99	30.0(15.0-41.7)		30.0(8.6-63.2)	
Female	176	32.5(11; 14)		20.5(14; 26)		84	30.0(8.6-30.0)		30.0(20.3-60.0)	
**Child’s age**			0.018		<0.001			0.868		0.922
≤ 2years	274	29.8(24; 35)		13.2(09; 17)		100	30.0(17.1-30.0)		30.0(17.1-60.0)	
3-5 years	138	42.0(34; 50)		31.9(24; 40)		83	30.0(8.6-38.6)		30.0(15.0-61.1)	
**Having siblings**			0.055		<0.001			0.972		0.205
Yes	158	39.9(32; 47)		31.0(24; 38)		88	30.0(8.6-38.6)		34.3(17.1-68.6)	
No	252	30.2(24; 36)		12.3(8; 16)		95	30.0(15.0-30.0)		30.0(12.9-45.0)	
**Having older siblings** ^§^			0.299		0.929			0.168		0.576
Yes	104	43.3(34; 53)		31.7(23; 41)		63	30.0(8.6-30.0)		30.0(17.1-60.0)	
No	54	33.3(21; 46)		29.6(17; 42)		25	30.0(22.5-54.6)		36.0(15.0-90.0)	
**Relationship between the respondent and the child** ^||^			0.045		0.008			0.755		0.608
Mother	378	32.3(27; 37)		17.7(14; 21)		163	30.0(8.6-36.4)		30.0(17.1-60.0)	
Father	28	53.6(35; 72)		39.3(21; 57)		17	25.7(8.6-49.3)		38.6(17.1-79.3)	
**Respondent’s age**			1.000		<0.001			0.159		0.175
≤35 years	220	34.1(28; 40)		12.3(8; 17)		89	30.0(17.1-38.6)		30.0(8.6-55.7)	
>35 years	190	33.7(27; 40)		27.9(21; 34)		94	30.0(8.6-30.0)		30.0(21.4-72.9)	
**Respondent’s educational level**			<0.001		0.144			0.584		0.832
non-University	54	64.8(52; 77)		27.8(16; 40)		40	30.0(21.4-34.3)		38.6(12.9-66.4)	
University	356	29.2(24 ;34)		18.3(14; 22)		143	30.0(8.6-38.6)		30.0(17.1-60.0)	

* p-values calculated with the Chi-square test

^†^ For the calculation of the time of use, children who did not use the devices were removed (n= 410 to n= 183)

^‡^ p-values calculated with the Mann-Whitney test

^§^ The n’s were calculated only for those who had siblings

^||^ The categories do not add up to 100 because data from “Others” were deleted

 An estimated 26.2% (95% CI: 20; 32) of the children who used any of the devices had free access to smartphone and/or tablet. Children with siblings had a significantly higher probability of having free access to smartphone and/or tablet compared to children without siblings (35.2% vs 17.9%, p-value=0.013) ( Table 2 ). 

**Table 2 -:** Overall percentage (%) of children with free access to smartphone and/or tablet (n=183) among those using devices and according to covariates. Spain, 2021-2022

	**n**	**Free access to smartphone and/or tablet**	**p-value**
		**Percentage** * **%(95% CI)**	
**Overall**	183	26.2(20; 32)	0.875
**Child’s gender**			
Male	99	25.3(17; 34)	
Female	84	27.4(18; 37)	
**Child’s age**			0.208
≤ 2 years	100	22.0(14; 30)	
3-5 years	83	31.3(21; 41)	
**Having siblings**			0.013
Yes	88	35.2(25; 45)	
No	95	17.9(10; 26)	
**Having older siblings** ^†^			1.000
Yes	63	34.9(23; 47)	
No	25	36.0(17; 55)	
**Relationship between the respondent and the child** ^‡^			0.333
Mother	163	25.8(19; 32)	
Father	17	23.5(17; 30)	
**Respondent’s age**			0.434
≤35 years	89	23.0(14; 32)	
>35 years	94	29.8(20; 39)	
**Respondent’s educational**			0.799
non-University	40	30.6(16; 45)	
University	143	25.2(18; 32)	

* p-values calculated with the Chi-square test

^†^ The n’s were calculated only for those who had siblings

^‡^ The categories do not add up to 183 because data from “Others” were deleted

 The most used applications among mobile and/or tablet users were video applications (75.4% (95% CI: 69; 82)) and a total of 36.1% (95% CI: 29; 43) of parents had applications on their mobile and/or tablet for the exclusive use of their child. In children up to 2 years of age, 20.0% (95% CI: 12; 28) use educational applications and 42.0% (95% CI: 32; 52) use music applications ( Table 3 ). In the 3 to 5 years age group, 41.0% (95% CI: 30; 52) used game applications. In terms of the type of app used exclusively, there were statistically significant differences between age groups in the proportion of games and educational use. 

**Table 3 -:** Overall percentage (%) of children using devices by type of application or application-only use, and according to groups of age. Spain, 2021-2022

	**Overall (n=183)**	**≤ 2 years (n=100)**	**3-5 years (n=83)**	**p-value** ^‡^
	**Percentage %(95% CI)** ^†^	**Percentage %(95% CI)** ^†^	**Percentage %(95% CI)** ^†^	
**Type of application your child uses** *				
Videos	138	68.0 (59; 77)	84.3 (76; 92)	0.017
	75.4 (69; 82)			
Games	44	10.0 (4; 15)	41.0 (30; 52)	<0.001
	24.0 (18; 30)			
Educational	61	20.0 (12; 28)	49.4 (39; 60)	<0.001
	33.3 (26; 40)			
Social Networking	2	1.0 (0; 3)	1.2 (-1; 3)	1.000
	1.1 (0;3)			
Music	63	42.0 (32; 52)	25.3 (16; 35)	0.038
	34.4 (27; 41)			
Other	11	8.0 (2; 13)	3.6 (0; 8)	0.352
	6.0 (2; 9)			
Not use	22	18.0 (10; 25)	4.8 (2; 9)	0.012
	12.0 (7; 17)			
**An application for the exclusive use of your child**				
Yes	66	21.0 (13; 29)	54.2 (43; 65)	<0.001
	36.1 (29; 43)			
No	117	79.0 (71; 87)	45.8 (35; 56)	
	63.9 (57; 70)			
**Type of application for exclusive use by children**				
Videos	45	18.0 (10; 25)	35.5 (25; 46)	0.036
	24.6 (18; 31)			
Games	35	7.0 (2; 12)	33.7 (25; 44)	<0.001
	19.1 (13; 25)			
Educational	39	11.0 (50; 17)	33.7 (25; 44)	<0.001
	21.3 (69; 82)			
Social Networking	1	0.0 (0; 0)	1.2 (-1; 3)	0.925
	0.5 (0; 1)			
Music	11	6.0 (1; 11)	6.0 (1;11)	1.000
	6.0 (2; 9)			
Other	3	2.0 (-1; 5)	1.2 (-1; 3)	1.000 ^§^
	1.6 (0; 3)			

* Each of the variables shown in the table are multiple response variables

^†^ Percentages (%) of columns do not add up to 100% because they are multiple choice

^‡^ p-values calculated with the Chi-square test

^§^ p-value calculated with the Z score test

 Among children who used smartphone and/or tablet, 11.6% (95% CI: 6; 16) used these devices up to 30 minutes after waking up during weekdays and 15.4% (95% CI: 10; 20) during weekend. Moreover, 14.0% (95% CI: 8; 19) used them up to 1 hour before going to the bed ( Table 4 ). 

**Table 4 -:** Overall percentage (%) of smartphone and/or tablet use upon waking up (weekday to weekend) and before going to bed and according to groups of age. Spain, 2021-2022

	**Percentage (%)**			
	**Overall (n=183)**	**≤ 2 years (n=100)**	**3-5 years (n=83)**	**p-value** *
**Use on waking up on weekdays**				0.945
5 minutes or less	3.3	3.0 (0; 6)	3.6 (0; 8)	
From 6 to 15 minutes	4.4	4.0 (0; 8)	4.8 (0; 9)	
From 16 to 30 minutes	3.8	3.0 (0; 6)	4.8 (0; 9)	
From 31 minutes to 1 hour	2.2	2.0 (-1; 4)	2.4 (0; 5)	
More than 1 hour	7.1	6.0 (1;11)	8.4 (2; 14)	
Not used	75.4	77.0 (69; 85)	73.5 (64; 83)	
**Use on waking up on weekends**				0.185
5 minutes or less	6.6	4.0 (0; 8)	9.6 (3; 16)	
From 6 to 15 minutes	4.4	5.0 (1; 9)	3.6 (0; 8)	
From 16 to 30 minutes	4.4	6.0 (1; 11)	2.4 (0; 5)	
From 31 minutes to 1 hour	2.2	1.0 (0; 3)	3.6 (0; 8)	
More than 1 hour	16.9	13.0 (6; 20)	21.7 (13; 30)	
Don’t know/ Don’t answer	61.2	65.0 (56; 74)	56.6 (46; 67)	
**Use it before going to bed**				0.599
Yes	14.8	13.0 (6; 20)	16.9 (9; 25)	
No	85.2	87.0 (80; 93)	83.1 (75; 91)	
**How long before going to bed**				0.683
30 minutes before	11.6	9.0 (3; 15)	14.5 (7; 22)	
1 hour before	2.4	2.0 (-1; 4)	2.4 (0; 5)	
More than 1 hour	0.6	1.0 (0; 3)	0.0 (0; 0)	
No use before going to bed	85.2	87.0 (80; 93)	83.1 (75; 91)	

* p-values calculated with the Fisher’s exact test and the Chi-square test

## Discussion

 To our knowledge, this is one of the first studies to characterize smartphones and tablets pattern of use in early childhood in Spain. Besides, our study is the first to discriminate the use of screens by type of device in Spain. The recommendations of health institutions do not consider the type of devices used (television, console, video game, smartphone, tablet, etc). The recommendations of health institutions - the MSSSI, World Health Organization, AAP and Canadian Pediatric Society - unanimously agreed that the time spent on devices and their use should be limited amongst the youngest of children. According to the 2017 National Health Survey of Spain (NHSS) ^(^ 13 ^)^ , among children aged 1 to 4 years, 57.8% spent an hour or more a day in front of a screen during the week and 62.9% on weekends. The proportion of those who reported spending an hour or more a day in front of a screen for recreational use increased with age, both during the week and on weekends. However, this survey in Spain estimates the global screen time during leisure time without separating according to type of device (e.g., TV, computer, console, smartphone, tablet). 

 62.3% of the world’s population uses social media and the mean daily usage is 2 hours and 23 minutes in January 2024 ^(^ 18 ^)^ . In our study, there are 75.4% (95% CI: 69; 82) of children who used video applications, and 36.1% (95% CI: 29; 43) of parents who had applications for exclusive use of their child. Importantly, the recommendations should discriminate between different types of screens, particularly smartphones, tablets, and TV due to the possible different effects of being exposed to these different devices. It is also important to make a record of quality multimedia applications to help parents to educate children to make a healthy use of smartphones and tablets during early childhood. Our study is in line with the NHSS ^(^ 13 ^)^ , in which the free time spent by the child population in front of a screen (for recreational purposes) was slightly higher on weekends than on weekdays. We found that 1 out of 10 children used the smartphone and/or tablet up to 30 minutes after waking up during weekdays, with higher percentage of use on weekends (11.5% vs 15.4% respectively). We believe that the use of devices shortly after awakening is an important indicator of dependence. On the one hand, we found that around 14.0% (95% CI: 8; 19) use smartphone and/or tablet up 1 hour before going to the bed. In this sense, previous studies have shown that using screen devices before going to bed affects the quality and time to fall sleep because blue light hinders the production of melatonin ^(^ 19 ^-^ 20 ^)^ . On the other hand, there is an association between the use of smartphones and tablets in the bedroom and delayed bedtime ^(^ 21 ^)^ . For this reason, the recommendation of pediatric guidelines should consider important aspects of using this type of smartphone and/or tablet such as using them few times after waking and before going to bed. 

 Owning one’s own smartphone in early childhood is widespread, being much higher than previously published data ^(^ 22 ^-^ 23 ^)^ , and in half of the children who use smartphones and/or tablets there is no adult control. In our study, within the group that has free access to the smartphone and/or tablet, 12.2% consider the smartphone theirs, and 36.4% consider the tablet theirs. By considering the device as his or her own, the child has the power to decide when and how to use it and it can encourage children’s autonomy and responsibility. However, it can also pose challenges in terms of setting appropriate limits and ensuring a balanced and safe use of the smartphone. Being in an older age group increases the likelihood of using a tablet, and having siblings is also associated with owning a tablet. The higher usage could be due to parents with older children being more permissive. Parents will be older and less energetic to play with their children and allow more use of smartphone and/or tablet. In responses from older parents there is a higher percentage of tablet use and free access to these devices. Nevertheless, in the case of smartphone this does not occur, we believe this could be because parents with fewer resources only have their smartphone as a device at home. 

 Smartphones and tablets are used by families to entertain their children and to calm them down, among others. However, the literature does not recommend excessive screen time use because they have a major impact on health ^(^ 24 ^)^ [worse sleep patterns ^(^ 25 ^)^ , increasing risk of obesity ^(^ 26 ^)^ and sedentary behavior ^(^ 7 ^)^ , worse cognitive development ^(^ 19 ^)^ (inattention and hyperactivity), worse language development and academic performance, and mental and emotional health ^(^ 20 ^)^ ]. In addition, our data are in line with a previous study ^(^ 27 ^)^ that children of parents with a lower education level are associated with a higher percentage of screen usage, however, our study differentiates between device types. In contrast to a previous study, we do not find any differences between smartphone nor tablet duration of usage, and the respondent’s education level. In this sense it is worth to mention that most of the parents included in our sample have a university degree. 

Finally, knowledge of the relationship between children’s possession of these devices and the time spent using them will help to guide valid intervention strategies to minimize the time spent using these devices by these vulnerable groups. In our opinion, we consider that daycare centers would be a good place to inform parents about the responsible use of screens with the goal of disseminate on social networks. Nurses and other healthcare would also be an essential figure to inform about the risk of higher exposure to screens. To this end, it is important to propose a good-practice guideline of screen time for different age groups since the different exposed effects vary according to age.

One of the most important study’s limitations is that our sample is not likely to be representative of the Spanish population due to the non-probabilistic sampling method used, which might limit the external validity of our conclusions.

The use of smartphones and tablets has been underestimated due to the overrepresentation of university studies (86.8%) in comparison with the study population. In addition, having collected the sample from social network profiles specialized in pediatrics could favor the entry of profiles of parents more concerned about the health and the lifestyle of their children and therefore also more informed.

Another limitation is derived from the use of an unvalidated, self-reported online questionnaire, which could introduce an information bias. Finally, the screen time variable was collected qualitatively. This may be another limitation, as we had to underestimate screen time responses when transforming this variable to quantitative.

 Summing up, our study provides relevant information for the lack of evidence of exposure to specific screen devices smartphone and/or tablet exclusively in Spain, being this type of devices the ones that are increasing among pediatric population ^(^ 28 ^)^ . 

## Conclusion

Around 50% of children used devices (smartphone and/or tablet) at least 30 minutes daily. High smartphone usage was associated to parents’ low education level and the child’s older age (3-5 years). Tablet usage correlated with having siblings, the child being older (3-5 years), and parents being over 35 years old. Screen time before bedtime should be considered in the new recommendations. Health professionals should advise against smartphone and tablet use in the hours before sleep and just after waking up. Nurses could provide educational guidelines, especially to parents, to regulate children’s screen devices use given the excessive use of screens in the pediatric population.
