# Supplementary material for: Smartphone and tablet use pattern in children up to 5 years old in Spain: a cross-sectional study
Source: Rev Lat Am Enfermagem. 2024 Nov 22;32:e4377. doi: 10.1590/1518-8345.7276.4377 (PMC11654049; doi:10.1590/1518-8345.7276.4377)
Supplement: Translation

# Translation: Patrón de uso de *smartphones* y *tablets* en niños de hasta 5 años en España: estudio transversal*

**DOI:** 10.1590/1518-8345.7276.4376

**Published:** 2024-11-15

**Authors:** Sonia de Paz-Cantos, Adrián González-Marrón, Cristina Lidón-Moyano, Maria Cerrato-Lara, Ana Díez-Izquierdo, Jose M Martínez-Sánchez

**Affiliations:** ^1^ Universitat Internacional de Catalunya, Barcelona, Sant Cugat del Vallès, España.; ^2^ Universidad Internacional de La Rioja, Logroño, España.

**Keywords:** Aplicaciones Móviles, Dispositivos Electrónicos, Preescolar, Promoción de la Salud, Smartphone, Tablet

## Abstract

caracterizar el patrón de uso de *smartphones* y *tablets* en una muestra de niños de hasta 5 años y evaluar con qué variables se asocia.

estudio transversal con 410 padres de niños de hasta 5 años de edad. Los datos se obtuvieron a través de un cuestionario *online* . Se realizaron preguntas sobre el tiempo y patrón de uso diario de *smartphones* y *tablets* . Se calculó el porcentaje global (y el intervalo de confianza del 95%) y la mediana (y rango intercuartílico) de tiempo de uso de cada dispositivo, según las variables asociadas al niño y a las características del entrevistado.

se estima que el 44,7% (IC 95%: 37; 51) de los niños utilizaban diariamente *smartphones* y/o *tablets* . El tiempo medio de uso de *smartphones* y *tablets* por separado fue de 30 minutos por día para los *smartphones* (8,6-38,6) y de 30 (17,1-60,0) para las *tablets* . El 11,6% (IC 95%: 6; 16) de los niños que tienen acceso libre a dispositivos los utiliza dentro de los 30 minutos después de haberse despertado durante la semana, el 15,4% (IC 95%: 10; 21) durante los fines de semana y el 14,0% (IC 95%: 8; 19) utiliza *smartphone* y/o *tablet* hasta 1 hora antes de dormir.

más de 4 de cada 10 niños de nuestra muestra utilizan *smartphones* y/o *tablets* diariamente. La enfermería comunitaria puede ser una opción para implementar programas educativos orientados a promover prácticas saludables sobre el uso de dispositivos electrónicos por parte de los niños, debido al patrón de uso excesivo de pantallas en la población pediátrica.

## Destacados

**(1)** Clasificar el uso de la pantalla según el lugar y el uso.

**(2)** El nivel educativo de los padres influye en el tiempo que los niños utilizan los dispositivos móviles.

**(3)** Hay que seguir las recomendaciones pediátricas.

**(4)** Los políticos, enfermeros y docentes deben trabajar juntos.

**(5)** Más de 4 de cada 10 niños de nuestra muestra utilizan *smartphones* y *tablets* diariamente.

## Introducción

 Los dispositivos con pantalla son omnipresentes y se utilizan para una variedad de tareas, que incluyen entretenimiento, comunicación y aprendizaje. Los niños y adolescentes están constantemente expuestos a una amplia variedad de dispositivos con pantalla ( *smartphones* , *tablets* y relojes inteligentes, entre otros) ^(^ 1 ^-^ 2 ^)^ . Por lo que aumentó el tiempo de uso de *smartphones* y *tablets* en los últimos años ^(^ 3 ^)^ . Además, el uso indiscriminado de las pantallas también ha invadido los hogares, provocando que los niños comiencen a utilizar medios digitales a edades cada vez más tempranas, entre los 12 y 24 meses de edad ^(^ 4 ^)^ . Por ejemplo, en Corea del Sur, el 12,2% de los niños empieza a utilizar medios digitales entre antes de los 6 meses y los 4 años; la mayoría de los niños de 3 y 4 años utilizan regularmente los teléfonos de sus padres sin ayuda, según una muestra de niños de Filadelfia ^(^ 5 ^)^ . 

 El uso de *smartphones* tiene efectos positivos y negativos sobre la salud ^(^ 6 ^)^ . Por un lado, el uso excesivo de *smartphones* y *tablets* (uso de pantallas durante más de 1 hora por día) se asocia con resultados perjudiciales para la salud de los niños en edad preescolar. Existe evidencia de que el tiempo frente a la pantalla está asociado con el sueño (especialmente con la duración reducida) y con el funcionamiento físico, mental y psicosocial de los bebés y niños pequeños ^(^ 7 ^-^ 8 ^)^ . Además, la obesidad es uno de los resultados asociados con la exposición a las pantallas mejor documentado ^(^ 9 ^-^ 10 ^)^ . Por otro lado, el uso de pantallas puede tener efectos positivos, como rápido acceso a la información, mayor comunicación e incluso aprendizaje educativo ^(^ 6 ^)^ . Los padres pueden introducir el uso de dispositivos con pantalla para ayudar a los niños con las tareas escolares. Además, hay evidencia preliminar de que las aplicaciones interactivas para “aprender a leer” y los libros electrónicos pueden promover la alfabetización temprana a través de la práctica del reconocimiento de letras, imágenes y palabras ^(^ 11 ^)^ . 

 Dada la creciente tendencia a utilizar *smartphones* y *tablets* a edades cada vez más tempranas, es importante realizar esfuerzos para evitar los efectos negativos del uso de las pantallas desde la perspectiva de la enfermería. El papel de la enfermería escolar puede ser muy importante para promover el uso saludable de las pantallas ^(^ 12 ^)^ . Los enfermeros son los profesionales de la salud que mayor contacto tienen con las familias en la atención primaria pediátrica. Por lo tanto, pueden liderar programas para promover el buen uso de la tecnología e incluso diseñar intervenciones para reducir el mal uso de *smartphones* . 

 Diferentes instituciones han hecho recomendaciones sobre el uso de los mismos. Según el Ministerio de Sanidad, Servicios Sociales e Igualdad (MSSSI) de España, los niños menores de 3 años no deben estar expuestos a pantallas y una exposición mayor a 1 hora diaria se considera excesiva para los niños de 2 a 4 años ^(^ 13 ^)^ . De forma similar, la Academia Estadounidense de Pediatría (AAP, siglas en inglés) recomienda que los niños de 18 a 24 meses estén expuestos solo a aplicaciones de alta calidad (por ejemplo, Duolingo) y que los niños de 3 a 5 años solo a 1 hora de pantalla con programas de alta calidad en presencia de los padres ^(^ 14 ^)^ , recomendación que adoptó recientemente el gobierno de Cataluña (España) ^(^ 15 ^)^ . Hasta la fecha no existen recomendaciones específicas sobre el tiempo de uso de *smartphones* y *tablets* . Además, la Encuesta Nacional de Salud de España informa el tiempo de exposición a pantallas en general, pero no realiza una distinción por dispositivo ^(^ 13 ^,^ 16 ^)^ . Por lo tanto, el objetivo de este estudio fue caracterizar el patrón de uso de *smartphones* y *tablets* en una muestra de niños de hasta 5 años y evaluar con qué variables se asocia. 

## Método

### Diseño del estudio

 Se realizó un estudio transversal desde marzo de 2021 a marzo de 2022. El informe de esta investigación se realizó siguiendo las directrices de la iniciativa STROBE ( *Strengthening the Reporting of Observational Studies in Epidemiology* ). 

### Configuración del estudio

 Utilizamos datos de referencia del proyecto *Smart Screen Health* , cuyo objetivo es promover el uso saludable de dispositivos con pantalla en la primera infancia. Desde *Kenko Lab* (centro de estudios sobre el uso saludable de pantallas durante la infancia en España), estamos realizando varios estudios para generar evidencia científica y soluciones que ayuden a las familias a crear buenos hábitos con la tecnología y encontrar formas de mejorar el desarrollo infantil ^(^ 17 ^)^ . 

 Los datos se recolectaron en el Hospital General de Catalunya, un centro médico de referencia de segundo nivel ubicado en la comunidad autónoma de Cataluña (España). Los investigadores distribuyeron en el ala principal de pediatría del Hospital General de Catalunya (HUGC) (Sant Cugat del Vallès, Barcelona, España) folletos que incluían el código de respuesta rápida (QR) vinculado a un cuestionario dirigido a los padres que acudían a la consulta pediátrica. Además, se colocaron dos carteles en la sala de espera del HUGC con el código QR para acceder directamente al cuestionario. También se utilizaron las cuentas del pediatra *influencer* “dospediatrasencasa” ( *Instagram* y *Twitter* ) para difundir el cuestionario entre los padres fuera del HUGC. 

### Población y muestra

Reclutamos una muestra de padres de niños españoles menores de 5 años. Se formó una muestra no probabilística y consecutiva de padres que asistieron a la sesión de estudio y que cumplían con los siguientes criterios de elegibilidad: padres que tenían niños de 3 meses a 5 años. Se obtuvieron 461 respuestas en total. De las 461 respuestas, se eliminaron 51 respuestas que presentaban errores (valores no plausibles) en las variables edad de los niños y edad de los padres. En definitiva, fueron excluidos 6 niños y 45 padres. Después de la limpieza de datos, la muestra final incluyó 410 respuestas.

### Tamaño de la muestra

El tamaño de la muestra se calculó mediante la fórmula de muestreo aleatorio simple (N = ([Z-p-(1-p)]/e)2) para una prevalencia estimada (p) del 50% (prevalencia que maximiza el tamaño de la muestra), un nivel de confianza del 95% (= 0,05; Z = 1,96) y una precisión o error del 5% (e = 0,05). Por consiguiente, eran necesarios 384 participantes.

### Instrumento de recolección de datos

 Se utilizó una encuesta *online* no validada para recolectar respuestas de padres de niños de entre 3 meses y 5 años de edad en España. El cuestionario *online* en español tenía 22 preguntas y fue implementado en *Google Forms* . El cuestionario incluía preguntas sociodemográficas **,** relacionadas con la salud y con la exposición a *smartphones* , *tablets* y TV. 

### Variables del estudio

Las variables del estudio incluyeron los siguientes resultados:

#### Uso de smartphone y/o tablet

 El uso estimado de *smartphones* y/o *tablets* se obtuvo por separado a través de dos preguntas: “¿Aproximadamente cuánto tiempo pasa su hijo frente a los siguientes dispositivos diariamente (de lunes a viernes)?” y “¿Aproximadamente cuánto tiempo pasa su hijo frente a los siguientes dispositivos los fines de semana (sábado y domingo)?”. Ambas preguntas incluían intervalos de 30 minutos (“0 minutos”, “30 minutos”, “1 hora”, “1 hora y 30 minutos”, “2 horas”, “2 horas y 30 minutos”, “3 horas”, “3 horas y 30 minutos”, “4 horas o más”). La opción “4 horas o más” se transformó en 4 horas (240 minutos) y se asumió que los niños expuestos a 4 horas o más de pantalla miraron exactamente 4 horas. Se consideraron usuarios los hijos de padres que informaron un uso de *smartphones* y *tablets* de al menos 30 minutos. Mientras que los niños con 0 minutos de uso fueron considerados no usuarios. 

#### Tiempo de uso diario de smartphone y/o tablet

 El uso diario de *smartphones* y/o *tablets* se evaluó mediante las preguntas mencionadas anteriormente en la sección uso de *smartphones* y/o *tablets* . Para obtener el tiempo promedio de uso diario se realizó un promedio ponderado de las respuestas a ambas preguntas de lunes a viernes y de sábado a domingo. Para calcular el tiempo de uso diario de cada dispositivo se ponderaron las respuestas de cada una de las dos preguntas multiplicándolas por 5 y 2, respectivamente. Estos resultados luego se sumaron y se dividieron por 7. 

#### Acceso libre al smartphone y/o tablet de algún miembro de la familia

 Para estimar el acceso libre a *smartphones* y/o *tablets* se formularon tres preguntas: “¿Su hijo tiene acceso libre a algún *smartphone* o *tablet* de algún miembro de la familia?” (“sí, no”); “¿A cuál de estos dispositivos?” (“ *smartphone* ”, “ *tablet* ”); “¿Su hijo considera que el *smartphone* / *tablet* es suyo?” 

#### Tipo de aplicación de uso y aplicación de uso exclusivo

 Para describir el tipo de aplicación que usa y el tipo de aplicación que usa exclusivamente, se formularon tres preguntas: “¿Qué tipo de aplicación utiliza su hijo frecuentemente?” (“vídeos”, “juegos”, “educativa”, “red social”, “música”, “otra”, “no usa”, “no sabe”/“no contesta”); “¿Tiene alguna aplicación en su *smartphone* para uso exclusivo de su hijo?” (“sí”, “no”, “no sabe”/“no contesta”); para aquellos que respondieron “sí” en la pregunta anterior, los padres podían responder “¿Cuál?” (“videos”, ” juegos”, “educativa”, “red social”, “música”, “otra”, “no usa”, “no sabe”/“no contesta”). Además, esas preguntas también se aplicaron para padres que respondieron que sus hijos estaban expuestos al dispositivo: ¿Su hijo usa su *smartphone* o *tablet* cuando se despierta? (“sí”, “no”). ¿Su hijo usa su *smartphone* o *tablet* antes de acostarse? (“sí”, “no”). Para estimar el tiempo que pasa usando el *smartphone* o la *tablet* después de despertarse durante la semana y los fines de semana: “¿Cuánto tiempo le toma a su hijo pedir/usar el *smartphone* después de despertarse los días que tiene que ir al colegio (de lunes a viernes)?”, “¿Cuánto tiempo le toma a su hijo pedir usar el *smartphone* después de despertarse? ¿Cuánto tiempo le toma a su hijo pedir usar el *smartphone* después de despertarse los fines de semana (sábado y domingo) o feriados?”, las dos preguntas tienen las mismas posibles respuestas (“no me lo pide”, “5 minutos o menos”, “6 a 15 minutos”, “16 a 30 minutos”, “31 minutos a una hora”, “más de 1 hora”, “no sabe”/“no contesta”). Para el estudio del uso de *smartphone* o *tablet* antes de acostarse: “¿Su hijo utiliza un *smartphone* o *tablet* antes de acostarse?” (“sí”, “no”, “no sabe/no contesta”), “¿Cuánto tiempo antes?” (“30 minutos antes”, “1 hora antes”, “más de 1 hora antes”, “no sabe/no contesta”). 

### Covariables

#### Variables asociadas al niño

Género (“Masculino”, “Femenino”); edad (“≤ 2 años”, “3-5 años”), tener hermanos (“sí”, “no”); y tener hermanos mayores (“sí”, “no”). Cuando tienen hermanos, también se preguntó si los hermanos eran mayores.

#### Variables asociadas al encuestado

Relación del entrevistado con el niño (“madre”, “padre”); edad (“≤ 35 años”, “>35 años”) y nivel educativo (“no universitario”, “universitario”).

### Análisis estadístico

 Se calculó el porcentaje de niños que utilizan un *smartphone* y/o *tablet* . Se calculó la mediana y el rango intercuartílico (RIC) del tiempo de uso diario por dispositivo ( *smartphone* y/o *tablet* ) para cada usuario de cada dispositivo. La mediana y el RIC del tiempo de uso se calcularon debido a la violación del supuesto de normalidad. Además, calculamos el porcentaje global de niños que utilizan un *smartphone* y/o *tablet* (n=183) con acceso libre al dispositivo y según las covariables. Se calcularon los porcentajes generales del tipo de aplicación y posesión de los padres de aplicaciones para uso exclusivo de sus hijos, estratificados por edad. También se calculó el porcentaje de exposición a *smartphones* y/o *tablets* al despertar y antes de acostarse de forma general y estratificada por edad. Para estudiar la asociación entre los resultados y las covariables se realizaron las pruebas de Mann-Whitney, Chi-Cuadrado y puntaje Z. El programa estadístico utilizado para realizar los análisis estadísticos fue el R-4.1.1. Se estableció un nivel de significación de 0,05. 

### Aspectos éticos

 Antes de iniciar el estudio, los participantes tuvieron acceso a la hoja informativa y al documento de consentimiento informado, que debían completar. Para aceptar responder la encuesta, los participantes completaron un formulario de consentimiento informado *online* , ya que todos los datos se tratan de forma anónima y únicamente con fines científicos. El presente estudio fue aprobado por el Comité de Ética en Investigación del HUGC, Barcelona (2021/09-PED-HUGC). 

## Resultados

 La muestra final incluyó información de 410 niños, de los cuales el 44,7% estuvo expuesto a *smartphones* y/o *tablets* ( Figura 1 ). El 57,0% de los niños era del sexo femenino, el 66,8% era menor de 3 años, el 86,8% de los padres tenía estudios universitarios, el 38,5% de los niños tenía hermanos y, de ellos, el 65,8% tenía hermanos mayores. 

**Figura 1:** - Diagrama de flujo de niños expuestos y no expuestos a dispositivos con pantalla en la muestra

 De los 410 niños, el 33,9% (IC 95%: 29; 38) utilizaba *smartphone* , el 19,6% (IC 95%: 15; 23) utilizaba *tablet* y el 44,7% (IC 95%: 37; 52) utilizaba ambos dispositivos. La mediana (P1-P3) del tiempo de uso diario de *smartphones* fue de 30,0 (8,6-38,6) minutos y la mediana de la *tablet* fue de 30,0 (17,1-60,0) minutos. La probabilidad de utilizar *smartphones* se asoció significativamente con el nivel educativo de los padres (valor p <0,001) y la edad del niño (valor p 0,018). En el caso de la *tablet* , la probabilidad de uso se asoció con la edad del niño, el hecho de tener hermanos y la edad de los entrevistados (p-valor <0,001) ( Tabla 1 ). 

**Tabla 1:** - Porcentaje (%) y mediana (y rango intercuartílico) de tiempo de uso de *smartphone* y/o *tablet* (en minutos) según las variables sociodemográficas de los niños y la familia. España, 2021-2022

	Smartphone **(n=139)**			Tablet **(n=80)**			Smartphone **(n=139)**		Tablet **(n=80)**	
	Porcentaje de uso (%)*						**Tiempo de uso (minutos por día)** ^‡^			
	**n**	**%(IC 95%)**	**p-valor**	**%(IC 95%)**	**p-valor**	**n** ^†^	**mediana (P1-P3)**	**p-valor**	**Mediana (P1-P3)**	p-valor*
**General**	410	33,9 (29; 38)		19,6 (15; 23)		183	30,0 (8,6-38,6)		30,0 (17,1-60,0)	
**Género del niño**			0,551		0,770			0,056		0,856
Masculino	234	35,8 (30; 41)		18,8 (14; 24)		99	30,0 (15,0-41,7)		30,0 (8,6-63,2)	
Femenino	176	32,5 (11; 14)		20,5 (14; 26)		84	30,0 (8,6-30,0)		30,0 (20,3-60,0)	
**Edad del niño**			0,018		<0,001			0,868		0,922
≤ 2 años	274	29,8 (24; 35)		13,2 (09; 17)		100	30,0 (17,1-30,0)		30,0 (17,1-60,0)	
3 a 5 años	138	42,0 (34; 50)		31,9 (24; 40)		83	30,0 (8,6-38,6)		30,0 (15,0-61,1)	
**Tener hermanos**			0,055		<0,001			0,972		0,205
Sí	158	39,9 (32; 47)		31,0 (24; 38)		88	30,0 (8,6-38,6)		34,3 (17,1-68,6)	
No	252	30,2 (24; 36)		12,3 (8; 16)		95	30,0 (15,0-30,0)		30,0 (12,9-45,0)	
**Tener hermanos mayores** ^§^			0,299		0,929			0,168		0,576
Sí	104	43,3 (34; 53)		31,7 (23; 41)		63	30,0 (8,6-30,0)		30,0 (17,1-60,0)	
No	54	33,3 (21; 46)		29,6 (17; 42)		25	30,0 (22,5-54,6)		36,0 (15,0-90,0)	
**Relación entre el entrevistado y el niño** ^||^			0,045		0,008			0,755		0,608
Madre	378	32,3 (27; 37)		17,7 (14; 21)		163	30,0 (8,6-36,4)		30,0 (17,1-60,0)	
Padre	28	53,6 (35; 72)		39,3 (21; 57)		17	25,7 (8,6-49,3)		38,6 (17,1-79,3)	
**Edad del encuestado**			1,000		<0,001			0,159		0,175
≤35 años	220	34,1 (28; 40)		12,3 (8; 17)		89	30,0 (17,1-38,6)		30,0 (8,6-55,7)	
>35 años	190	33,7 (27; 40)		27,9 (21; 34)		94	30,0 (8,6-30,0)		30,0 (21,4-72,9)	
**Nivel educativo del encuestado**			<0,001		0,144			0,584		0,832
No Universitario	54	64,8 (52; 77)		27,8 (16; 40)		40	30,0 (21,4-34,3)		38,6 (12,9-66,4)	
Universitario	356	29,2 (24 ;34)		18,3 (14; 22)		143	30,0 (8,6-38,6)		30,0 (17,1-60,0)	

* Valores p calculados con la prueba de chi-cuadrado

^†^ Para calcular el tiempo de uso se excluyó a los niños que no usaron los dispositivos (n= 410 para n = 183)

^‡^ Valores de p calculados con la prueba de Mann-Whitney

^§^ Las n se calcularon sólo para aquellos que tenían hermanos

^||^ Las categorías no suman 100 porque se excluyeron “Otros” datos

 Se estima que el 26,2% (IC 95%: 20; 32) de los niños que utilizaban alguno de los dispositivos tenían acceso libre a un *smartphone* y/o *tablet* . Los niños con hermanos tenían significativamente más probabilidades de tener acceso libre a un *smartphone* y/o *tablet* que los niños sin hermanos (35,2% vs. 17,9%, valor p=0,013) ( Tabla 2 ). 

**Tabla 2:** - Porcentaje general (%) de niños con acceso libre a un *smartphone* y/o *tablet* (n=183) entre los que utilizan dispositivos y en función de las covariables. España, 2021-2022

	**n**	**Acceso libre al** *smartphone* **y/o** *tablet*	p-valor*
		Porcentaje* %(IC 95%)	
**General**	183	26,2 (20; 32)	
**Género del niño**			0,875
Masculino	99	25,3 (17; 34)	
Femenino	84	27,4 (18; 37)	
**Edad del niño**			0,208
≤ 2 años	100	22,0 (14; 30)	
3 a 5 años	83	31,3 (21; 41)	
**Tener hermanos**			0,013
Sí	88	35,2 (25; 45)	
No	95	17,9 (10; 26)	
**Tener hermanos mayores** ^†^			1,000
Sí	63	34,9 (23; 47)	
No	25	36,0 (17; 55)	
**Relación entre el entrevistado y el niño** ^‡^			0,333
Madre	163	25,8 (19; 32)	
Padre	17	23,5 (17; 30)	
**Edad del encuestado**			0,434
≤35 años	89	23,0 (14; 32)	
>35 años	94	29,8 (20; 39)	
**Nivel educativo del encuestado**			0,799
No Universitario	40	30,6 (16; 45)	
Universitario	143	25,2 (18; 32)	

* Valores p calculados con la prueba de chi-cuadrado

^†^ Las n se calcularon sólo para aquellos que tenían hermanos

^‡^ Las categorías no suman 183 porque se excluyeron “Otros” datos

 Las aplicaciones más utilizadas por los usuarios de *smartphones* y/o *tablets* eran aplicaciones de vídeo (75,4% (IC 95%: 69; 82)) y el 36,1% (IC 95%: 29; 43) de los padres tenían aplicaciones en sus *smartphones* y/o *tablets* para uso exclusivo de sus hijos. El 20,0% (IC 95%: 12; 28) de los niños de hasta 2 años de edad utilizaba aplicaciones educativas y el 42% (IC 95%: 32; 52) utilizaba aplicaciones musicales ( Tabla 3 ). En el grupo etario de 3 a 5 años, el 41,0% (IC 95%: 30; 52) utilizaba aplicaciones de juegos. En cuanto al tipo de aplicación que usan exclusivamente, hubo diferencias estadísticamente significativas entre los grupos etarios en la proporción de juegos y educativas. 

**Tabla 3 -:** Porcentaje general (%) de niños que utilizan dispositivos por tipo de aplicación o uso exclusivo de aplicaciones y por grupo etario. España, 2021-2022

	**General (n=183)**	**≤ 2 años (n=100)**	**3-5 años (n=83)**	**p-valor** ^‡^
	**Porcentaje %(IC 95%)** ^†^	**Porcentaje %(IC 95%)** ^†^	**Porcentaje %(IC 95%)** ^†^	
Tipo de aplicación que usa su hijo*				
Videos	138	68,0 (59; 77)	84,3 (76; 92)	0,017
	75,4 (69; 82)	10,0 (4; 15)	41,0 (30; 52)	<0,001
Juegos	44			
	24,0 (18; 30)	20,0 (12; 28)	49,4 (39; 60)	<0,001
Educativa	61			
	33,3 (26; 40)	1,0 (0; 3)	1,2 (-1; 3)	1,000
Redes sociales	2			
	1,1 (0;3)	42,0 (32; 52)	25,3 (16; 35)	0,038
Música	63			
	34,4 (27; 41)	8,0 (2; 13)	3,6 (0; 8)	0,352
Otras	11			
	6,0 (2; 9)	18,0(10; 25)	4,8(2; 9)	0,012
No usa	22 12,0(7; 17)			
**Tiene una aplicación para uso exclusivo de su hijo**		21,0 (13; 29)	54,2 (43; 65)	<0,001
Sí	66			
	36,1 (29; 43)	79,0 (71; 87)	45,8 (35; 56)	
No	117			
	63,9 (57; 70)			
**Tipo de aplicación para uso exclusivo de los niños**		18,0 (10; 25)	35,5 (25; 46)	0,036
Videos	45			
	24,6 (18; 31)	7,0 (2; 12)	33,7 (25; 44)	<0,001
Juegos	35			
	19,1 (13; 25)	11,0 (50; 17)	33,7 (25; 44)	<0,001
Educativa	39			
	21,3 (69; 82)	0,0 (0; 0)	1,2 (-1; 3)	0,925
Redes sociales	1			
	0,5 (0; 1)	6,0 (1; 11)	6,0 (1;11)	1,000
Música	11			
	6,0 (2; 9)	2,0 (-1; 5)	1,2 (-1; 3)	1,000 ^§^
Otras	3 1,6 (0; 3)			

* Cada una de las variables que se muestran en la tabla es una variable de respuesta múltiple

^†^ Los porcentajes (%) de las columnas no suman 100% porque son de opción múltiple

^‡^ Valores p calculados con la prueba de chi-cuadrado

^§^ Valor p calculado con la prueba de puntaje Z

 El 11,6% (IC 95%: 6; 16) de los niños que usaban un *smartphone* y/o *tablet* , usaba estos dispositivos dentro de los 30 minutos después de haberse despertado durante la semana y el 15,4% (IC 95%: 10; 20) durante el fin de semana. Además, el 14,0% (IC 95%: 8; 19) los utilizaba hasta 1 hora antes de acostarse ( Tabla 4 ). 

**Tabla 4 -:** Porcentaje general (%) de uso de *smartphone* y/o *tablet* al despertarse (de lunes a viernes a fin de semana) y antes de acostarse, según el grupo etario. España, 2021-2022

	**Porcentaje (%)**			
	**General (n=183)**	**≤ 2 años (n=100)**	**3-5 años (n=83)**	p-valor*
**Uso al despertarse durante la semana**				0,945
5 minutos o menos	3,3	3,0 (0; 6)	3,6 (0; 8)	
De 6 a 15 minutos	4,4	4,0 (0; 8)	4,8 (0; 9)	
De 16 a 30 minutos	3,8	3,0 (0; 6)	4,8 (0; 9)	
De 31 minutos a 1 hora	2,2	2,0 (-1; 4)	2,4 (0; 5)	
Más de 1 hora	7,1	6,0 (1;11)	8,4 (2; 14)	
No usa	75,4	77,0 (69; 85)	73,5 (64; 83)	
**Uso al despertarse los fines de semana**				0,185
5 minutos o menos	6,6	4,0 (0; 8)	9,6 (3; 16)	
De 6 a 15 minutos	4,4	5,0 (1; 9)	3,6 (0; 8)	
De 16 a 30 minutos	4,4	6,0 (1; 11)	2,4 (0; 5)	
De 31 minutos a 1 hora	2,2	1,0 (0; 3)	3,6 (0; 8)	
Más de 1 hora	16,9	13,0 (6; 20)	21,7 (13; 30)	
No sabe / No contesta	61,2	65,0 (56; 74)	56,6 (46; 67)	
**Uso antes de irse a dormir**				0,599
Sí	14,8	13,0 (6; 20)	16,9 (9; 25)	
No	85,2	87,0 (80; 93)	83,1 (75; 91)	
**Cuanto tiempo antes de acostarse**				0,683
30 minutos antes	11,6	9,0 (3; 15)	14,5 (7; 22)	
1 hora antes	2,4	2,0 (-1; 4)	2,4 (0; 5)	
Más de 1 hora	0,6	1,0 (0; 3)	0,0 (0; 0)	
No usa antes de acostarse	85,2	87,0 (80; 93)	83,1 (75; 91)	

* Valores de p calculados con la prueba exacta de Fisher y la prueba de chi-cuadrado

## Discusión

 Hasta donde sabemos, este es uno de los primeros estudios que caracteriza el patrón de uso de *smartphones* y *tablets* en la primera infancia en España. Además, nuestro estudio es el primero que clasifica el uso de pantalla por tipo de dispositivo en España. Las recomendaciones de las instituciones de salud no consideran el tipo de dispositivo utilizado (televisión, consola, videojuego, *smartphone* , *tablet* , etc.). Las recomendaciones de las instituciones de salud (MSSSI, Organización Mundial de la Salud, AAP y Sociedad Canadiense de Pediatría) coincidieron de forma unánime en que hay que limitar el tiempo que le dedican los niños a los dispositivos y el uso por parte de los niños más pequeños. Según la Encuesta Nacional de Salud de España (ENSE) de 2017 ^(^ 13 ^)^ , el 57,8% de los niños entre 1 y 4 años pasaba una hora o más al día frente a una pantalla durante la semana y el 62,9 % los fines de semana. La proporción de los que pasaban una hora o más al día frente a una pantalla para uso recreativo aumentó con la edad, tanto durante la semana como los fines de semana. Sin embargo, esa investigación en España calcula el tiempo total frente a una pantalla durante el tiempo libre sin clasificarlo por tipo de dispositivo (por ejemplo, TV, computadora, videojuego, *smartphone* , *tablet* ). 

 El 62,3% de la población mundial utilizaba las redes sociales y el uso diario promedio era de 2 horas y 23 minutos en enero de 2024 ^(^ 18 ^)^ . En nuestro estudio, el 75,4% (IC 95%: 69; 82) de los niños utilizaba aplicaciones de vídeo y el 36,1% (IC 95%: 29; 43) de los padres tenía aplicaciones para uso exclusivo de sus hijos. Es importante destacar que las recomendaciones deben discriminar entre diferentes tipos de pantallas, especialmente *smartphones* , *tablets* y TV, debido a que es posible que tengan diferentes efectos producto de la exposición a los diferentes dispositivos. También es importante hacer una lista de las aplicaciones multimedia de calidad para ayudar a los padres a educar a los niños en el uso saludable de los *smartphones* y las *tablets* durante la primera infancia. Nuestro estudio coincide con la (ENSE) ^(^ 13 ^)^ en que el tiempo libre que la población infantil dedica frente a una pantalla (con fines recreativos) es ligeramente mayor los fines de semana que los días laborables. Comprobamos que 1 de cada 10 niños utilizó su *smartphone* y/o *tablet* dentro de los 30 minutos después de despertarse durante los días laborables, y que el porcentaje de uso fue mayor los fines de semana (11,5% vs. 15,4%, respectivamente). Creemos que el uso de dispositivos poco después de despertarse es un indicador importante de adicción. Por un lado, descubrimos que alrededor del 14,0% (IC 95%: 8; 19) utiliza un *smartphone* y/o *tablet* hasta 1 hora antes de acostarse. Hay estudios previos que demostraron que el uso de dispositivos con pantalla antes de acostarse afecta la calidad y el tiempo del sueño, ya que la luz azul dificulta la producción de melatonina ^(^ 19 ^-^ 20 ^)^ . Por otro lado, existe una asociación entre el uso de *smartphones* y *tablets* en el dormitorio y dormirse tarde ^(^ 21 ^)^ . Por ello, las recomendaciones de las guías pediátricas deben considerar aspectos importantes en el uso de *smartphones* y/o *tablets* , como usarlos pocas veces después de despertarse y antes de acostarse. 

 Tener un *smartphone* propio en la primera infancia está muy extendido, con valores muy superiores a los datos publicados anteriormente ^(^ 22 ^-^ 23 ^)^ y la mitad de los niños que utilizan *smartphones* y/o *tablets* no son supervisados por adultos. En nuestro estudio, en el grupo que tiene acceso libre a un *smartphone* y/o *tablet* , el 12,2% considera que el smartphone le pertenece y el 36,4% considera que la *tablet* es suya. Cuando el niño considera que el dispositivo le pertenece, tiene el poder de decidir cuándo y cómo utilizarlo y esto puede fomentar la autonomía y la responsabilidad en los niños. Sin embargo, esto también puede presentar desafíos para establecer límites adecuados y garantizar un uso equilibrado y seguro de los *smartphones* . Formar parte de un grupo etario de mayor edad aumenta la probabilidad de utilizar una *tablet* , y tener hermanos también se asocia con poseer una *tablet* . Un mayor uso puede deberse a que los padres con hijos mayores son más permisivos. Los padres tendrán mayor edad y menos energía para jugar con sus hijos y les permitirán usar más *smartphones* y/o *tablets* . En las respuestas de los padres con mayor edad se observa un mayor porcentaje de uso de *tablets* y acceso libre a estos dispositivos. Pero eso no sucede con los *smartphones* . Creemos que esto puede deberse a que el único dispositivo que hay en casa de los padres con menos recursos es el *smartphone* . 

 Las familias utilizan los *smartphones* y las *tablets* para entretener a los niños y calmarlos, entre otras cosas. Sin embargo, la literatura no recomienda el uso excesivo de pantallas porque tiene un gran impacto en la salud ^(^ 24 ^)^ [peores patrones de sueño ^(^ 25 ^)^ , mayor riesgo de obesidad ^(^ 26 ^)^ , comportamiento sedentario ^(^ 7 ^)^ , peor desarrollo cognitivo ^(^ 19 ^)^ (falta de atención e hiperactividad), peor desarrollo del lenguaje y rendimiento académico, y salud mental y emocional ^(^ 20 ^)^ ]. Además, nuestros datos coinciden con los de un estudio previo ^(^ 27 ^)^ según el cual los hijos de padres con menor nivel educativo se asocian a un mayor porcentaje de uso de pantallas, pero nuestro estudio distingue los tipos de dispositivos. A diferencia de los resultados que arrojó un estudio anterior, no encontramos diferencias entre la duración del uso de *smartphones* y *tablets* y el nivel educativo de los encuestados. Por otro lado, cabe mencionar que la mayoría de los padres incluidos en nuestra muestra tienen título universitario. 

Por último, saber cuál es la relación entre que los niños sean dueños de los dispositivos y el tiempo que pasan usándolos ayudará a orientar estrategias de intervención válidas para reducir el tiempo que estos grupos vulnerables dedican a usar dichos dispositivos. En nuestra opinión, consideramos que las guarderías serían un buen lugar para informarles a los padres sobre el uso responsable de las pantallas, con el objetivo de difundir la información en las redes sociales. Los enfermeros y otros profesionales de la salud también serían una figura fundamental para informar sobre el riesgo de una mayor exposición a las pantallas. Para ello, es importante proponer una guía de buenas prácticas de tiempo frente a la pantalla para diferentes grupos etarios, ya que los efectos de la exposición varían según la edad.

Una de las limitaciones más importantes del estudio es que nuestra muestra probablemente no sea representativa de la población española, porque se utilizó un método de muestreo no probabilístico, lo que puede limitar la validez externa de nuestras conclusiones.

 El uso de *smartphones* y *tablets* fue subestimado debido a la sobrerrepresentación de los estudios universitarios (86,8%) en comparación con la población de estudio. Además, el hecho de que la muestra se haya recolectado a partir de perfiles de redes sociales especializadas en pediatría podría favorecer la inclusión de perfiles de padres más preocupados por la salud y el estilo de vida de sus hijos y, por lo tanto, más informados. 

 Otra limitación es el uso de un cuestionario *online* no validado y autoadministrado, que podría introducir sesgos de información. Por último, se recogió cualitativamente la variable tiempo frente a la pantalla. Esta puede ser otra limitación, ya que tuvimos que subestimar las respuestas sobre el tiempo frente a la pantalla al transformar esta variable en cuantitativa. 

 En síntesis, nuestro estudio aporta información relevante a la escasa evidencia que hay sobre exposición a dispositivos con pantalla específica, *smartphones* y/o *tablets* , exclusivamente en España, ya que es este tipo de dispositivos el que está aumentando en la población pediátrica ^(^ 28 ^)^ . 

## Conclusión

 Alrededor del 50% de los niños utilizaban dispositivos ( *smartphone* y/o *tablet* ) al menos 30 minutos por día. El elevado uso de *smartphones* se asoció con el bajo nivel educativo de los padres y la edad avanzada del niño (3 a 5 años). El uso de *tablets* se asoció con tener hermanos, tener más edad (3 a 5 años) y que los padres tuvieran más de 35 años. Hay que considerar el tiempo frente a la pantalla antes de acostarse en nuevas recomendaciones. Los profesionales de la salud deberían desaconsejar el uso de *smartphones* y *tablets* horas antes de dormir y poco después de despertarse. Los enfermeros podrían brindar orientación educativa, especialmente a los padres, para regular el uso de dispositivos con pantalla por parte de los niños, debido al uso excesivo de pantallas que registra la población pediátrica. 

# Translation: Padrão de uso de *smartphones* e *tablets* em crianças de até cinco anos de idade na Espanha: um estudo transversal *

**DOI:** 10.1590/1518-8345.7276.4378

**Published:** 2024-11-15

**Authors:** Sonia de Paz-Cantos, Adrián González-Marrón, Cristina Lidón-Moyano, Maria Cerrato-Lara, Ana Díez-Izquierdo, Jose M Martínez-Sánchez

**Affiliations:** ^1^ Universitat Internacional de Catalunya, Barcelona, Sant Cugat del Vallès, Espanha.; ^2^ Universidad Internacional de La Rioja, Logroño, Espanha.

**Keywords:** Aplicativos Móveis, Dispositivos Eletrônicos, Pré-escolar, Promoção da Saúde, Smartphone, Tablet

## Abstract

caracterizar o padrão de uso de *smartphones* e *tablets* em uma amostra de crianças de até cinco anos de idade e avaliar com quais variáveis ele está associado.

estudo transversal com 410 pais de crianças de até cinco anos de idade. Os dados foram obtidos por meio de um questionário *online* . Foram coletadas perguntas sobre o tempo e o padrão de uso diário de *smartphones* e *tablets* . Calculamos a porcentagem geral (e o intervalo de confiança de 95%) e a mediana (e o intervalo interquartil) do tempo de uso de cada dispositivo, de acordo com as variáveis associadas à criança e as características do entrevistado.

estima-se que 44,7% (IC 95%: 37; 51) das crianças usavam *smartphones* e/ou *tablets* diariamente. O tempo médio de uso de *smartphones* e *tablets,* separadamente, foi de 30 minutos por dia para *smartphones* (8,6-38,6) e 30 (17,1-60,0) para *tablets* . Nas crianças que têm acesso livre aos dispositivos, 11,6% (IC 95%: 6; 16) usam esses dispositivos até 30 minutos depois de acordar durante a semana, 15,4% (IC 95%: 10; 21) durante os finais de semana e 14,0% (IC 95%: 8; 19) usam *smartphone* e/ou *tablet* até 1 hora antes de dormir.

mais de quatro em cada dez crianças de nossa amostra usavam *smartphones* e/ou *tablets* diariamente. A enfermagem comunitária pode ser um caminho viável para a implementação de programas educacionais voltados para a promoção de práticas saudáveis no uso de dispositivos eletrônicos por crianças, dado o padrão de uso excessivo de telas na população pediátrica.

## Destaques

**(1)** Separar o uso da tela de acordo com o local e o uso.

**(2)** O nível educacional dos pais influencia o tempo de uso de dispositivos móveis pelas crianças.

**(3)** As recomendações pediátricas devem ser seguidas.

**(4)** Políticos, enfermeiros e professores devem trabalhar juntos.

**(5)** Mais de quatro em cada dez crianças de nossa amostra usam *smartphones* e *tablets* diariamente.

**Todos os autores aprovaram a versão final do texto.**

* A publicação deste artigo na Série Temática “Saúde digital: contribuições da enfermagem” se insere na atividade 2.2 do Termo de Referência 2 do Plano de Trabalho do Centro Colaborador da OPAS/OMS para o Desenvolvimento da Pesquisa em Enfermagem, Brasil. Apoio financeiro do Ministerio de Ciencias e Innovación do Governo da Espanha, processo nº 2021SGR00186, Espanha.

**Objetivo** : caracterizar o padrão de uso de *smartphones* e *tablets* em uma amostra de crianças de até cinco anos de idade e avaliar com quais variáveis ele está associado. **Método** : estudo transversal com 410 pais de crianças de até cinco anos de idade. Os dados foram obtidos por meio de um questionário *online* . Foram coletadas perguntas sobre o tempo e o padrão de uso diário de *smartphones* e *tablets* . Calculamos a porcentagem geral (e o intervalo de confiança de 95%) e a mediana (e o intervalo interquartil) do tempo de uso de cada dispositivo, de acordo com as variáveis associadas à criança e as características do entrevistado. **Resultados** : estima-se que 44,7% (IC 95%: 37; 51) das crianças usavam *smartphones* e/ou *tablets* diariamente. O tempo médio de uso de *smartphones* e *tablets,* separadamente, foi de 30 minutos por dia para *smartphones* (8,6-38,6) e 30 (17,1-60,0) para *tablets* . Nas crianças que têm acesso livre aos dispositivos, 11,6% (IC 95%: 6; 16) usam esses dispositivos até 30 minutos depois de acordar durante a semana, 15,4% (IC 95%: 10; 21) durante os finais de semana e 14,0% (IC 95%: 8; 19) usam *smartphone* e/ou *tablet* até 1 hora antes de dormir. **Conclusão** : mais de quatro em cada dez crianças de nossa amostra usavam *smartphones* e/ou *tablets* diariamente. A enfermagem comunitária pode ser um caminho viável para a implementação de programas educacionais voltados para a promoção de práticas saudáveis no uso de dispositivos eletrônicos por crianças, dado o padrão de uso excessivo de telas na população pediátrica. 

## Introdução

 Os dispositivos de tela são onipresentes e usados para várias tarefas, incluindo entretenimento, comunicação e aprendizado. As crianças e os adolescentes são constantemente expostos a uma grande variedade de dispositivos de tela, incluindo ( *smartphones* , *tablets* e *smartwatches* , entre outros) ^(^ 1 ^-^ 2 ^)^ . Consequentemente, o tempo de uso de *smartphones* e *tablets* aumentou nos últimos anos ^(^ 3 ^)^ . Além disso, o uso indiscriminado de telas também invadiu os lares, fazendo com que as crianças comecem a usar mídia digital em idades cada vez menores, entre 12 e 24 meses de idade ^(^ 4 ^)^ . Por exemplo, na Coreia do Sul, 12,2% das crianças começam a usar mídia digital antes dos seis meses até os quatro anos de idade; a maioria das crianças de três e quatro anos acessa regularmente os telefones dos pais sem qualquer assistência, de acordo com uma amostra de crianças da Filadélfia ^(^ 5 ^)^ . 

 O uso de *smartphones* tem efeitos positivos e negativos sobre a saúde ^(^ 6 ^)^ . Por um lado, o uso excessivo de *smartphone* e *tablet* (uso da tela por mais de uma hora por dia) está associado a resultados de saúde prejudiciais em crianças pré-escolares. Há evidências de que o tempo de tela está associado ao sono (principalmente com duração reduzida) e ao funcionamento físico, mental e psicossocial de bebês e crianças pequenas ^(^ 7 ^-^ 8 ^)^ . Além disso, a obesidade é um dos resultados mais bem documentados associados à exposição à tela ^(^ 9 ^-^ 10 ^)^ . Por outro lado, o uso de telas pode ter efeitos positivos, como acesso rápido a informações, maior comunicação e até mesmo aprendizado educacional ^(^ 6 ^)^ . Os pais podem introduzir dispositivos de tela para ajudar as crianças com as tarefas escolares. Além disso, há evidências preliminares de que aplicativos interativos “aprendendo a ler” e livros eletrônicos podem promover a alfabetização precoce por meio da prática de reconhecimento de letras, imagens e palavras ^(^ 11 ^)^ . 

 Dada a tendência crescente do uso de *smartphones* e *tablets* em idades cada vez mais jovens, é importante fazer esforços para evitar os efeitos negativos do uso de telas do ponto de vista da enfermagem. O papel da enfermagem escolar pode ser muito importante na promoção do uso saudável das telas ^(^ 12 ^)^ . Os enfermeiros são os profissionais de saúde que têm o maior contato com as famílias na atenção primária pediátrica. Portanto, eles podem liderar programas para promover o bom uso da tecnologia e até mesmo projetar intervenções para reduzir o uso indevido de *smartphones* . 

 Diferentes instituições fizeram recomendações sobre seu uso. De acordo com o Ministério da Saúde, Serviços Sociais e Igualdade ( *Ministerio de Sanidad, Servicios Sociales e Igualdad* : MSSSI, da Espanha), crianças com menos de três anos de idade não devem ser expostas a telas, e o tempo de tela de mais de 1 hora por dia é considerado excessivo entre crianças de dois a quatro anos ^(^ 13 ^)^ . Da mesma forma, a Academia Americana de Pediatria (AAP) recomenda que as crianças de 18 a 24 meses sejam expostas somente a aplicativos de alta qualidade (por exemplo, Duolingo) e que as crianças de três a cinco anos sejam limitadas a uma hora de tempo de tela com programas de alta qualidade na presença dos pais ^(^ 14 ^)^ , recomendação recentemente adotada pelo governo da Catalunha (Espanha) ^(^ 15 ^)^ . Até o momento, não há recomendações específicas sobre o tempo de uso de *smartphones* e *tablets* . Além disso, a Pesquisa Nacional de Saúde da Espanha relata a exposição ao tempo de tela em geral, sem diferenciação por dispositivo ^(^ 13 ^,^ 16 ^)^ . Portanto, o objetivo deste estudo foi caracterizar o padrão de uso de *smartphones* e *tablets* em uma amostra de crianças de até cinco anos de idade e avaliar com quais variáveis ele está associado. 

## Método

### Desenho do estudo

 Um estudo transversal foi realizado de março de 2021 a março de 2022. Esta pesquisa foi relatada de acordo com a diretriz STROBE ( *Strengthening the Reporting of Observational Studies in Epidemiology* ). 

### Configuração do estudo

 Usamos dados de linha de base do projeto *Smart Screen Health* , que visa promover o uso saudável de dispositivos de tela na primeira infância. A partir do *Kenko Lab* (um centro de estudos para o uso saudável da tela durante a infância na Espanha), estamos realizando vários estudos para gerar evidências científicas e soluções que ajudem as famílias a criar bons hábitos com a tecnologia e encontrar maneiras de melhorar o desenvolvimento infantil ^(^ 17 ^)^ . 

 Os dados foram coletados no *Hospital General de Catalunya* , um centro médico de referência de segundo nível localizado na comunidade autônoma da Catalunha (Espanha). Os pesquisadores distribuíram na ala pediátrica principal do *Hospital Universitario General de Catalunya* (HUGC) (Sant Cugat del Vallès, Barcelona, Espanha) folhetos que incluíam o código de resposta rápida (QR) vinculado a um questionário para os pais que estavam participando das consultas pediátricas. Além disso, dois pôsteres foram afixados na sala de espera do HUGC com o QR para acessar diretamente o questionário. Além disso, as contas de um pediatra influenciador “dospediatrasencasa” ( *Instagram* e *Twitter* ) também foram usadas para divulgar o questionário para os pais fora do HUGC. 

### População e amostra

Recrutamos uma amostra de pais de crianças espanholas com menos de cinco anos de idade. Uma amostra não probabilística e consecutiva foi formada por pais que compareceram à sessão de estudo e que atenderam aos seguintes critérios de elegibilidade: eram pais que tinham filhos de três meses a cinco anos de idade. Foi obtido um total de 461 respostas. Dessas 461 respostas, foram eliminadas 51 respostas que apresentavam erros (valores não plausíveis) nas variáveis de idade das crianças e idade dos pais. Especificamente, foram eliminadas seis crianças e 45 pais. Após a limpeza dos dados, a amostra final incluiu 410 respostas.

### Tamanho da amostragem

O tamanho da amostra foi calculado com a fórmula de amostragem aleatória simples (N = ([Z-p-(1-p)]/e)2) para uma prevalência estimada (p) de 50% (prevalência que maximiza o tamanho da amostra), um nível de confiança de 95% (= 0,05; Z = 1,96) e uma precisão ou erro de 5% (e = 0,05). De acordo com isso, foram necessários 384 indivíduos.

### Instrumento de coleta de dados

 Uma pesquisa *online* não validada foi usada para coletar respostas de pais com filhos entre três meses e cinco anos de idade na Espanha. O questionário *online* em espanhol tinha 22 perguntas e foi implementado no *Google Forms* . O questionário incluía perguntas relacionadas à saúde, sociodemográficas e à exposição a *smartphones* , *tablets* e TV. 

### Variáveis do estudo

As variáveis do estudo incluíram os resultados abaixo:

#### Uso de smartphone e/ou tablet

 A estimativa de uso de *smartphone* e/ou *tablet* foi obtida separadamente por meio de duas perguntas: “Aproximadamente, quanto tempo seu filho passa na frente dos seguintes dispositivos diariamente (de segunda a sexta-feira)?” e “Aproximadamente, quanto tempo seu filho passa na frente dos seguintes dispositivos nos fins de semana (sábado e domingo)?”. Ambas foram solicitadas em intervalos de 30 minutos (“0 minutos”, “30 minutos”, “1 hora”, “1 hora e 30 minutos”, “2 horas”, “2 horas e 30 minutos”, “3 horas”, “3 horas e 30 minutos”, “4 horas ou mais”). “4 horas ou mais” foi transformado em 4 horas (240 minutos) e presumiu-se que as crianças expostas a quatro horas ou mais de tempo de tela assistiram exatamente quatro horas. Os filhos de pais que relataram pelo menos 30 minutos de uso de *smartphone* e *tablet* foram considerados usuários. Caso contrário, as crianças que relataram zero minutos de uso foram consideradas não usuárias. 

#### Tempo de uso diário do smartphone e/ou tablet

 O uso diário do *smartphone* e/ou *tablet* foi avaliado por meio das perguntas mencionadas acima na seção de uso do *smartphone* e/ou *tablet* . Para obter o tempo médio de uso diário, foi realizada a média ponderada das respostas a ambas as perguntas de segunda a sexta-feira e de sábado a domingo. Para estimar o tempo de uso diário de cada dispositivo, as respostas a cada uma das duas perguntas foram ponderadas, multiplicando-as por cinco e por dois, respectivamente. Esses resultados foram então somados e divididos por sete. 

#### Acesso gratuito a qualquer smartphone e/ou tablet de qualquer membro da família

 Para estimar o acesso livre ao *smartphone* e/ou *tablet* , foram feitas três perguntas: “Seu filho tem acesso livre a qualquer *smartphone* ou *tablet* de qualquer membro da família?” (“sim”, “não”); “A quais desses dispositivos?” (“ *smartphone* ”, “ *tablet* ”); “Seu filho considera o *smartphone/tablet* como seu próprio?”. 

#### Tipo de aplicação de uso e aplicação de uso exclusivo

 Para descrever o tipo de aplicativo usado e o tipo de aplicativo de uso exclusivo, foram feitas três perguntas: “Que tipo de aplicativo seu filho usa com frequência?” (“vídeos”, “jogos”, “educacional”, “rede social”, “música”, “outro”, “não uso”, “não sei”/“sem resposta”); “Você tem algum aplicativo em seu *smartphone* para uso exclusivo de seu filho?” (“sim”, ” não”, “não sei”/“sem resposta”); para aqueles que responderam “sim” na questão anterior, os pais poderiam responder “Qual?” (“vídeos”, “jogos”, “educacional”, “rede social”, “música”, “outro”, “não uso”, “não sei”/“não respondeu”). Além disso, essas perguntas também foram usadas para os pais que responderam que seus filhos estavam expostos ao dispositivo: Seu filho usa o *smartphone* ou *tablet* ao acordar? (“sim”, “não”). Seu filho usa o *smartphone* ou *tablet* antes de ir para a cama? (“sim”, “não”). Duas perguntas foram usadas para estimar o tempo gasto usando o *smartphone* ou *tablet* depois de acordar nos dias de semana e nos finais de semana: “Quanto tempo seu filho leva para pedir/usar o *smartphone* depois de acordar nos dias de aula (segunda a sexta-feira)?”, “Quanto tempo seu filho leva para pedir para usar o *smartphone* depois de acordar nos finais de semana (sábado e domingo) ou feriados?”, ambas com as possíveis respostas (“nenhuma solicitação”, “5 minutos ou menos”, “6 a 15 minutos”, “16 a 30 minutos”, “31 minutos a uma hora”, “mais de 1 hora”, “não sei”/“não respondeu”). Para o estudo do uso de *smartphone* ou *tablet* antes de ir para a cama: “Seu filho usa *smartphone* ou *tablet* antes de ir para a cama?” (“sim”, “não”, “não sabe/não respondeu”), “Quanto tempo antes?” (“30 minutos antes”, “1 hora antes”, “mais de 1 hora antes”, “não sei/não respondeu”). 

### Covariáveis

#### Variáveis associadas à criança

Gênero (“masculino”, “feminino”); idade (“≤ 2 anos”, “3-5 anos”), ter irmãos (“sim”, “não”); e ter irmãos mais velhos (“sim”, “não”). Para aqueles que têm irmãos, também foi coletado se os irmãos eram mais velhos.

#### Variáveis associadas ao respondente

Relacionamento do entrevistado com a criança (“mãe”, “pai”); idade (“≤ 35 anos”, “>35 anos”) e nível educacional (“não universitário”, “universitário”).

### Análise estatística

 Foi calculada a porcentagem de crianças que usam *smartphone* e/ou *tablet* . A mediana e o intervalo interquartil (IQR) do tempo de uso diário por dispositivo ( *smartphone* e/ou *tablet* ) foram calculados para cada usuário de cada dispositivo. A mediana e o IQR do tempo de uso foram calculados devido à violação do pressuposto de normalidade. Além disso, calculamos a porcentagem geral de crianças que usam *smartphone* e/ou *tablet* (n=183) com acesso gratuito ao dispositivo e de acordo com as covariáveis. Foram calculadas as porcentagens gerais do tipo de aplicativo e da posse pelos pais de aplicativos exclusivos para uso dos filhos, estratificados por idade. Além disso, a porcentagem de exposição ao *smartphone* e/ou *tablet* ao acordar e antes de dormir foi calculada em geral e estratificada por idade. Para estudar a associação entre os resultados e as covariáveis, foram realizados os testes de Mann-Whitney, Qui-Quadrado e escore Z. O programa estatístico usado para realizar as análises estatísticas foi o R-4.1.1. O nível de significância foi estabelecido em 0,05. 

### Aspectos éticos

 Antes de iniciar o estudo, os participantes tiveram acesso à folha de informações e ao documento de consentimento informado, que tiveram de preencher. Para concordar em responder à pesquisa, os participantes preencheram um consentimento informado *online* , pois todos os dados são tratados de forma anônima e somente para fins científicos. O presente estudo foi aprovado pelo Comitê de Ética em Pesquisa do HUGC, Barcelona (2021/09-PED-HUGC). 

## Resultados

 A amostra final incluiu informações de 410 crianças, das quais 44,7% foram expostas a *smartphones* e/ou *tablets* (Figura 1). 57,0% das crianças eram do sexo feminino, 66,8% tinham menos de três anos de idade, 86,8% dos pais tinham estudos universitários, 38,5% das crianças tinham irmãos e, desses, 65,8% tinham irmãos mais velhos. 

**Figura 1 -:** Fluxograma de crianças expostas e não expostas a dispositivos de tela na amostra

 Das 410 crianças, 33,9% (IC 95%: 29; 38) usavam *smartphone* , 19,6% (IC 95%: 15; 23) usavam *tablet* e 44,7% (IC 95%: 37; 52) usavam ambos os dispositivos. A mediana (Q1-Q3) do tempo diário de uso do *smartphone* foi de 30,0 (8,6-38,6) minutos e 30,0 (17,1-60,0) minutos para o *tablet* . A probabilidade de uso de *smartphones* foi significativamente associada ao nível de escolaridade dos pais (p-valor <0,001) e à idade da criança (p-valor 0,018). No caso do *tablet* , a probabilidade de uso foi associada à idade da criança, ao fato de ter irmãos e à idade dos entrevistados (p-valor <0,001) (Tabela 1). 

**Tabela 1 -:** Porcentagem (%) e mediana (e intervalo interquartil) do tempo de uso (em minutos) do *smartphone* e/ou *tablet* de acordo com as variáveis sociodemográficas das crianças e da família. Espanha, 2021-2022

	Smartphone **(n=139)**			Tablet **(n=80)**			Smartphone **(n=139)**		Tablet **(n=80)**	
	Porcentagem de uso (%)*						**Tempo de uso (minutos por dia)** ^‡^			
	**n**	**%(IC 95%)**	**p-valor**	**%(IC 95%)**	**p-valor**	**n** ^†^	**mediana (Q1-Q3)**	**p-valor**	**Mediana (Q1-Q3)**	p-value*
**Geral**	410	33,9 (29; 38)		19,6 (15; 23)		183	30,0 (8,6-38,6)		30,0 (17,1-60,0)	
**Gênero da criança**			0,551		0,770			0,056		0,856
Masculino	234	35,8 (30; 41)		18,8 (14; 24)		99	30,0 (15,0-41,7)		30,0 (8,6-63,2)	
Feminino	176	32,5 (11; 14)		20,5 (14; 26)		84	30,0 (8,6-30,0)		30,0 (20,3-60,0)	
**Idade da criança**			0,018		<0,001			0,868		0,922
≤ 2 anos	274	29,8 (24; 35)		13,2 (09; 17)		100	30,0 (17,1-30,0)		30,0 (17,1-60,0)	
3 a 5 anos	138	42,0 (34; 50)		31,9 (24; 40)		83	30,0 (8,6-38,6)		30,0 (15,0-61,1)	
**Ter irmãos**			0,055		<0,001			0,972		0,205
Sim	158	39,9 (32; 47)		31,0 (24; 38)		88	30,0 (8,6-38,6)		34,3 (17,1-68,6)	
Não	252	30,2 (24; 36)		12,3 (8; 16)		95	30,0 (15,0-30,0)		30,0 (12,9-45,0)	
**Ter irmãos mais velhos** ^§^			0,299		0,929			0,168		0,576
Sim	104	43,3 (34; 53)		31,7 (23; 41)		63	30,0 (8,6-30,0)		30,0 (17,1-60,0)	
Não	54	33,3 (21; 46)		29,6 (17; 42)		25	30,0 (22,5-54,6)		36,0 (15,0-90,0)	
**Relacionamento entre o entrevistado e a criança** ^||^			0,045		0,008			0,755		0,608
Mãe	378	32,3 (27; 37)		17,7 (14; 21)		163	30,0 (8,6-36,4)		30,0 (17,1-60,0)	
Pai	28	53,6 (35; 72)		39,3 (21; 57)		17	25,7 (8,6-49,3)		38,6 (17,1-79,3)	
**Idade do entrevistado**			1,000		<0,001			0,159		0,175
≤35 anos	220	34,1 (28; 40)		12,3 (8; 17)		89	30,0 (17,1-38,6)		30,0 (8,6-55,7)	
>35 anos	190	33,7 (27; 40)		27,9 (21; 34)		94	30,0 (8,6-30,0)		30,0 (21,4-72,9)	
**Nível educacional do entrevistado**			<0,001		0,144			0,584		0,832
Não Universitário	54	64,8 (52; 77)		27,8 (16; 40)		40	30,0 (21,4-34,3)		38,6 (12,9-66,4)	
Universitário	356	29,2 (24 ;34)		18,3 (14; 22)		143	30,0 (8,6-38,6)		30,0 (17,1-60,0)	

* Valores p calculados com o teste qui-quadrado

^†^ Para o cálculo do tempo de uso, as crianças que não usaram os dispositivos foram removidas (n= 410 para n= 183)

^‡^ Valores p calculados com o teste Mann-Whitney

^§^ Os n’s foram calculados somente para aqueles que tinham irmãos

^||^ As categorias não somam 100 porque os dados de “Outros” foram excluídos

 Estima-se que 26,2% (IC 95%: 20; 32) das crianças que usaram qualquer um dos dispositivos tinham acesso livre a *smartphone* e/ou *tablet* . As crianças com irmãos tinham uma probabilidade significativamente maior de ter acesso livre a *smartphone* e/ou *tablet* em comparação com as crianças sem irmãos (35,2% vs. 17,9%, p-valor=0,013) (Tabela 2). 

**Tabela 2 -:** Porcentagem geral (%) de crianças com acesso livre a *smartphone* e/ou *tablet* (n=183) entre as que usam dispositivos e de acordo com covariáveis. Espanha, 2021-2022

	**n**	**Acesso livre ao** *smartphone* **e/ou** *tablet*	p-valor*
		Porcentagem* %(IC 95%)	
**Geral**	183	26,2 (20; 32)	
**Gênero da criança**			0,875
Masculino	99	25,3 (17; 34)	
Feminino	84	27,4 (18; 37)	
**Idade da criança**			0,208
≤ 2 anos	100	22,0 (14; 30)	
3 a 5 anos	83	31,3 (21; 41)	
**Ter irmãos**			0,013
Sim	88	35,2 (25; 45)	
Não	95	17,9 (10; 26)	
**Ter irmãos mais velhos** ^†^			1,000
Sim	63	34,9 (23; 47)	
Não	25	36,0 (17; 55)	
**Relacionamento entre o entrevistado e a criança** ^‡^			0,333
Mãe	163	25,8 (19; 32)	
Pai	17	23,5 (17; 30)	
**Idade do entrevistado**			0,434
≤35 anos	89	23,0 (14; 32)	
>35 anos	94	29,8 (20; 39)	
**Escolaridade do entrevistado**			0,799
Não Universitário	40	30,6 (16; 45)	
Universitário	143	25,2 (18; 32)	

* Valores p calculados com o teste de qui-quadrado

^†^ Os n’s foram calculados somente para aqueles que tinham irmãos

^‡^ As categorias não somam 183 porque os dados de “Outros” foram excluídos

 Os aplicativos mais usados entre os usuários de *smartphones* e/ou *tablets* foram os aplicativos de vídeo (75,4% (IC 95%: 69; 82)) e um total de 36,1% (IC 95%: 29; 43) dos pais tinham aplicativos em seus *smartphones* e/ou *tablets* para uso exclusivo de seus filhos. Em crianças de até 2 anos de idade, 20,0% (IC 95%: 12; 28) usam aplicativos educacionais e 42,0% (IC 95%: 32; 52) usam aplicativos de música (Tabela 3). Na faixa etária de três a cinco anos, 41,0% (IC 95%: 30; 52) usavam aplicativos de jogos. Em termos do tipo de aplicativo usado exclusivamente, houve diferenças estatisticamente significativas entre as faixas etárias na proporção de jogos e uso educacional. 

**Tabela 3 -:** Porcentagem geral (%) de crianças que usam dispositivos por tipo de aplicativo ou uso somente de aplicativo, e de acordo com grupos de idade. Espanha, 2021-2022

	**Geral (n=183)**	**≤ 2 anos (n=100)**	**3-5 anos (n=83)**	**p-valor** ^‡^
	**Porcentagem %(IC 95%)** ^†^	**Porcentagem %(IC 95%)** ^†^	**Porcentagem %(IC 95%)** ^†^	
Tipo de aplicativo que seu filho usa*				
Vídeos	138	68,0 (59; 77)	84,3 (76; 92)	0,017
	75,4 (69; 82)			
Jogos	44	10,0 (4; 15)	41,0 (30; 52)	<0,001
	24,0 (18; 30)			
Educacional	61	20,0 (12; 28)	49,4 (39; 60)	<0,001
	33,3 (26; 40)			
Redes sociais	2	1,0 (0; 3)	1,2 (-1; 3)	1,000
	1,1 (0;3)			
Música	63	42,0 (32; 52)	25,3 (16; 35)	0,038
	34,4 (27; 41)			
Outros	11	8,0 (2; 13)	3,6 (0; 8)	0,352
	6,0 (2; 9)			
Não usar	22	18,0 (10; 25)	4,8 (2; 9)	0,012
	12,0 (7; 17)			
**Um aplicativo para uso exclusivo de seu filho**				
Sim	66	21,0 (13; 29)	54,2 (43; 65)	<0,001
	36,1 (29; 43)			
Não	117	79,0 (71; 87)	45,8 (35; 56)	
	63,9 (57; 70)			
**Tipo de aplicativo para uso exclusivo de crianças**				
Vídeos	45	18,0 (10; 25)	35,5 (25; 46)	0,036
	24,6 (18; 31)			
Jogos	35	7,0 (2; 12)	33,7 (25; 44)	<0,001
	19,1 (13; 25)			
Educacional	39	11,0 (50; 17)	33,7 (25; 44)	<0,001
	21,3 (69; 82)			
Redes sociais	1	0,0 (0; 0)	1,2 (-1; 3)	0,925
	0,5 (0; 1)			
Música	11	6,0 (1; 11)	6,0 (1;11)	1,000
	6,0 (2; 9)			
Outros	3	2,0 (-1; 5)	1,2 (-1; 3)	1,000 ^§^
	1,6 (0; 3)			

* Cada uma das variáveis mostradas na tabela é uma variável de resposta múltipla

^†^ As porcentagens (%) das colunas não somam 100% porque são de múltipla escolha

^‡^ Valores p calculados com o teste de qui-quadrado

^§^ Valor p calculado com o teste de escore Z

 Entre as crianças que usavam *smartphone* e/ou *tablet* , 11,6% (IC 95%: 6; 16) usavam esses dispositivos até 30 minutos depois de acordar durante a semana e 15,4% (IC 95%: 10; 20) durante o fim de semana. Além disso, 14,0% (IC 95%: 8; 19) os utilizavam até uma hora antes de ir para a cama (Tabela 4). 

**Tabela 4 -:** Porcentagem geral (%) de uso de *smartphone* e/ou *tablet* ao acordar (de dia de semana a fim de semana) e antes de ir para a cama, de acordo com grupos de idade. Espanha, 2021-2022

	**Porcentagem (%)**			
	**Geral (n=183)**	**≤ 2 anos (n=100)**	**3-5 anos (n=83)**	p-valor*
**Uso ao acordar nos dias de semana**				0,945
5 minutos ou menos	3,3	3,0 (0; 6)	3,6 (0; 8)	
De 6 a 15 minutos	4,4	4,0 (0; 8)	4,8 (0; 9)	
De 16 a 30 minutos	3,8	3,0 (0; 6)	4,8 (0; 9)	
De 31 minutos a 1 hora	2,2	2,0 (-1; 4)	2,4 (0; 5)	
Mais de 1 hora	7,1	6,0 (1;11)	8,4 (2; 14)	
Não utilizado	75,4	77,0 (69; 85)	73,5 (64; 83)	
**Uso ao acordar nos finais de semana**				0,185
5 minutos ou menos	6,6	4,0 (0; 8)	9,6 (3; 16)	
De 6 a 15 minutos	4,4	5,0 (1; 9)	3,6 (0; 8)	
De 16 a 30 minutos	4,4	6,0 (1; 11)	2,4 (0; 5)	
De 31 minutos a 1 hora	2,2	1,0 (0; 3)	3,6 (0; 8)	
Mais de 1 hora	16,9	13,0 (6; 20)	21,7 (13; 30)	
Não sei/Não respondo	61,2	65,0 (56; 74)	56,6 (46; 67)	
**Uso antes de ir para a cama**				0,599
Sim	14,8	13,0 (6; 20)	16,9 (9; 25)	
Não	85,2	87,0 (80; 93)	83,1 (75; 91)	
**Quanto tempo antes de ir para a cama**				0,683
30 minutos antes	11,6	9,0 (3; 15)	14,5 (7; 22)	
1 hora antes	2,4	2,0 (-1; 4)	2,4 (0; 5)	
Mais de 1 hora	0,6	1,0 (0; 3)	0,0 (0; 0)	
Não usar antes de ir para a cama	85,2	87,0 (80; 93)	83,1 (75; 91)	

* Valores p calculados com o teste exato de Fisher e o teste do qui-quadrado

## Discussão

 Até onde sabemos, este é um dos primeiros estudos a caracterizar o padrão de uso de *smartphones* e *tablets* na primeira infância na Espanha. Além disso, nosso estudo é o primeiro a discriminar o uso de telas por tipo de dispositivo na Espanha. As recomendações das instituições de saúde não consideram o tipo de dispositivo usado (televisão, console, videogame, *smartphone* , *tablet* etc.). As recomendações das instituições de saúde - MSSSI, Organização Mundial da Saúde, AAP e Sociedade Canadense de Pediatria - concordaram unanimemente que o tempo gasto com dispositivos e seu uso deve ser limitado entre as crianças mais novas. De acordo com a Pesquisa Nacional de Saúde da Espanha (NHSS) de 2017 ^(^ 13 ^)^ , entre crianças de um a quatro anos, 57,8% passavam uma hora ou mais por dia em frente a uma tela durante a semana e 62,9% nos fins de semana. A proporção dos que relataram passar uma hora ou mais por dia em frente a uma tela para uso recreativo aumentou com a idade, tanto durante a semana quanto nos finais de semana. No entanto, essa pesquisa na Espanha estima o tempo global de tela durante o tempo de lazer sem separar de acordo com o tipo de dispositivo (por exemplo, TV, computador, videogame, *smartphone* , *tablet* ). 

 Na população mundial, 62,3% usam mídia social e o uso médio diário é de duas horas e 23 minutos em janeiro de 2024 ^(^ 18 ^)^ . Em nosso estudo, 75,4% (IC 95%: 69; 82) das crianças usavam aplicativos de vídeo e 36,1% (IC 95%: 29; 43) dos pais tinham aplicativos para uso exclusivo de seus filhos. É importante ressaltar que as recomendações devem discriminar entre diferentes tipos de telas, especialmente *smartphones* , *tablets* e TV, devido aos possíveis efeitos diferentes da exposição a esses diferentes dispositivos. Também é importante fazer um registro de aplicativos multimídia de qualidade para ajudar os pais a educar as crianças a fazer uso saudável de *smartphones* e *tablets* durante a primeira infância. Nosso estudo está de acordo com o NHSS ^(^ 13 ^)^ , segundo o qual o tempo livre gasto pela população infantil em frente a uma tela (para fins recreativos) foi ligeiramente maior nos fins de semana do que nos dias úteis. Constatamos que uma em cada dez crianças usou o *smartphone* e/ou *tablet* até 30 minutos após acordar durante os dias de semana, com maior porcentagem de uso nos fins de semana (11,5% vs. 15,4%, respectivamente). Acreditamos que o uso de dispositivos logo após o despertar é um importante indicador de dependência. Por um lado, descobrimos que cerca de 14,0% (IC 95%: 8; 19) usam *smartphone* e/ou *tablet* até uma hora antes de ir para a cama. Nesse sentido, estudos anteriores mostraram que o uso de dispositivos com tela antes de ir para a cama afeta a qualidade e o tempo de sono, pois a luz azul dificulta a produção de melatonina ^(^ 19 ^-^ 20 ^)^ . Por outro lado, há uma associação entre o uso de *smartphones* e *tablets* no quarto e o atraso na hora de dormir ^(^ 21 ^)^ . Por esse motivo, a recomendação de diretrizes pediátricas deve considerar aspectos importantes do uso desse tipo de *smartphone* e/ou *tablet* , como usá-los poucas vezes depois de acordar e antes de ir para a cama. 

 A posse do próprio *smartphone* na primeira infância é generalizada, sendo muito maior do que os dados publicados anteriormente ^(^ 22 ^-^ 23 ^)^ , e em metade das crianças que usam *smartphones* e/ou *tablets* não há controle de um adulto. Em nosso estudo, no grupo que tem acesso livre ao *smartphone* e/ou *tablet* , 12,2% consideram o *smartphone* como seu e 36,4% consideram o *tablet* como seu. Ao considerar o dispositivo como seu, a criança tem o poder de decidir quando e como usá-lo e isso pode incentivar a autonomia e a responsabilidade das crianças. No entanto, isso também pode representar desafios em termos de definição de limites adequados e de garantia de um uso equilibrado e seguro do *smartphone* . Estar em uma faixa etária mais velha aumenta a probabilidade de usar um *tablet* , e ter irmãos também está associado ao fato de possuir um *tablet* . O maior uso pode ser devido ao fato de os pais com filhos mais velhos serem mais permissivos. Os pais serão mais velhos e terão menos energia para brincar com seus filhos e permitirão mais uso do *smartphone* e/ou *tablet* . Nas respostas dos pais mais velhos, há uma porcentagem maior de uso do *tablet* e de acesso livre a esses dispositivos. No entanto, no caso do *smartphone* , isso não ocorre. Acreditamos que isso pode ser devido ao fato de os pais com menos recursos terem apenas o *smartphone* como dispositivo em casa. 

*Smartphones* e *tablets* são usados pelas famílias para entreter as crianças e acalmá-las, entre outros. No entanto, a literatura não recomenda o uso excessivo de tempo de tela porque eles têm um grande impacto na saúde ^(^ 24 ^)^ [piores padrões de sono ^(^ 25 ^)^ , aumento do risco de obesidade ^(^ 26 ^)^ , comportamento sedentário ^(^ 7 ^)^ , pior desenvolvimento cognitivo ^(^ 19 ^)^ (desatenção e hiperatividade), pior desenvolvimento da linguagem e desempenho acadêmico, e saúde mental e emocional ^(^ 20 ^)^ ]. Além disso, nossos dados estão de acordo com um estudo anterior ^(^ 27 ^)^ segundo o qual filhos de pais com nível de escolaridade mais baixo estão associados a uma porcentagem maior de uso de tela; no entanto, nosso estudo diferencia os tipos de dispositivos. Em contraste com um estudo anterior, não encontramos diferenças entre a duração do uso de *smartphones* e *tablets* e o nível de escolaridade do entrevistado. Nesse sentido, vale a pena mencionar que a maioria dos pais incluídos em nossa amostra tem diploma universitário. 

Por fim, o conhecimento da relação entre a posse desses dispositivos pelas crianças e o tempo gasto com eles ajudará a orientar estratégias de intervenção válidas para minimizar o tempo gasto com esses dispositivos por esses grupos vulneráveis. Em nossa opinião, consideramos que as creches seriam um bom lugar para informar os pais sobre o uso responsável das telas, com o objetivo de divulgá-las nas redes sociais. Os enfermeiros e outros profissionais de saúde também seriam uma figura essencial para informar sobre o risco de maior exposição às telas. Para isso, é importante propor uma diretriz de boas práticas de tempo de tela para diferentes faixas etárias, já que os diferentes efeitos expostos variam de acordo com a idade.

Uma das limitações mais importantes do estudo é que nossa amostra provavelmente não é representativa da população espanhola devido ao método de amostragem não probabilístico usado, o que pode limitar a validade externa de nossas conclusões.

 O uso de *smartphones* e *tablets* foi subestimado devido à super-representação de estudos universitários (86,8%) em comparação com a população do estudo. Além disso, o fato de a amostra ter sido coletada de perfis de redes sociais especializados em pediatria poderia favorecer a entrada de perfis de pais mais preocupados com a saúde e o estilo de vida de seus filhos e, portanto, também mais informados. 

 Outra limitação é derivada do uso de um questionário *online* não validado e autorrelatado, que poderia introduzir um viés de informação. Por fim, a variável de tempo de tela foi coletada qualitativamente. Isso pode ser outra limitação, pois tivemos de subestimar as respostas sobre o tempo de tela ao transformar essa variável em quantitativa. 

 Resumindo, nosso estudo fornece informações relevantes para a falta de evidências de exposição a dispositivos de tela específicos, *smartphone* e/ou *tablet* , exclusivamente na Espanha, sendo esse tipo de dispositivo o que está aumentando entre a população pediátrica ^(^ 28 ^)^ . 

## Conclusão

 Cerca de 50% das crianças usavam dispositivos ( *smartphone* e/ou *tablet* ) pelo menos 30 minutos por dia. O alto uso de *smartphones* foi associado ao baixo nível de escolaridade dos pais e à idade avançada da criança (três a cinco anos). O uso de *tablets* foi associado ao fato de ter irmãos, de a criança ser mais velha (três a cinco anos) e de os pais terem mais de 35 anos de idade. O tempo de tela antes de dormir deve ser considerado nas novas recomendações. Os profissionais de saúde devem desaconselhar o uso de *smartphones* e *tablets* nas horas que antecedem o sono e logo depois de acordar. Os enfermeiros poderiam fornecer orientações educacionais, especialmente aos pais, para regular o uso de dispositivos de tela das crianças, dado o uso excessivo de telas na população pediátrica.
